# Delphi consensus on Thoroughbred yearling sales endoscopy in Australasia

**DOI:** 10.1002/evj.70164

**Published:** 2026-04-14

**Authors:** Josephine L. Hardwick, Benjamin J. Ahern, Brian H. Anderson, Samantha H. Franklin

**Affiliations:** ^1^ School of Animal And Veterinary Sciences Adelaide University Adelaide South Australia Australia; ^2^ School of Veterinary Medicine Murdoch University Perth Western Australia Australia; ^3^ School of Veterinary Science University of Queensland Brisbane Queensland Australia; ^4^ Ballarat Veterinary Practice Ballarat Victoria Australia

**Keywords:** clinical guidelines, consensus, horse, sales, upper airway

## Abstract

**Background:**

Concerns regarding the reliability and consistency of yearling sales endoscopy in Australia and New Zealand have led to reduced industry confidence. Recent studies have clarified the relationship between yearling laryngeal function (YLF) grades and future outcomes.

**Objectives:**

To build expert consensus on the most appropriate method for grading YLF, assess the clinical relevance of each grade on future race performance and prosthetic laryngoplasty risk, and develop guidelines for pre‐sale endoscopic technique.

**Study Design:**

Modified Delphi study.

**Methods:**

Anonymous, iterative surveys were distributed to expert veterinarians in Australia and New Zealand. Consensus was defined a priori as ≥75% agreement. Surveys included closed and open‐ended questions, with qualitative and quantitative data analysed after each round. Panellists received a summary of responses before each subsequent round. Items reaching consensus were excluded from later rounds, and new items were added based on panellist feedback.

**Results:**

Three survey rounds were completed, with 40 veterinarians, 39 (97.5%) and 37 (92.3%) respectively. Consensus was achieved on adopting the Havemeyer grading system, standardising pre‐sale endoscopic techniques, and implementing a four‐tier risk‐rating system for YLF grades. Grades I and II.1 YLF were classified as low‐risk; grade II.2 as low‐moderate‐risk; grade III.1 as moderate‐risk; and ≥grade III.2 as high‐risk. Descriptors were developed to guide risk categorisation based on observed YLF.

**Main Limitations:**

Steering committee members were involved in prior research.

**Conclusions:**

This study provides a foundation for evidence‐based guidelines that enhance the transparency, consistency, and animal welfare in yearling sales endoscopy, supporting better‐informed purchasing decisions.

## Zusammenfassung

**Hintergrund:**

Bedenken hinsichtlich der Zuverlässigkeit und Konsistenz der Endoskopie bei Jährlingsauktionen in Australien und Neuseeland haben zu einem Rückgang des Vertrauens in die Branche geführt. Jüngste Studien legten den Zusammenhang zwischen Jährlings‐Kehlkopffunktion (YLF) und zukünftigen Ergebnissen dar.

**Ziel der Studie:**

Die Erreichung eines Expertenkonsens über die am besten geeignete Methode zur Einstufung von YLF, Bewertung der klinischen Relevanz jeden Grades für die zukünftige Leistungsfähigkeit und Risiko der Notwendigkeit einer prosthetischen Laryngoplastik, sowie Entwicklung von Richtlinien für die endoskopische Technik vor Auktion.

**Studiendesign:**

Modifizierte Delphi‐Studie.

**Methodik:**

Anonyme, interative Umfragen wurden an erfahrene Tierärzte (“Experten”) in Australien und Neuseeland verbreitet. Ein Konsens wurde a priori als eine Übereinstimmung von ≥75% definiert. Die Umfragen umfassten geschlossene und offene Fragen, wobei nach jeder Runde qualitative und quantitative Daten analysiert wurden. Die Panelteilnehmer erhielten vor jeder weiteren Runde eine Zusammenfassung der Antworten. Punkte, über die Einigkeit herrschte, wurden aus späteren Runden ausgeschlossen und auf Grundlage des Feedbacks der Panelteilnehmer wurden neue Punkte hinzugefügt.

**Ergebnisse:**

Es wurden drei Umfragerunden durchgeführt, an denen 40, 39 (97.5%) bzw. 37 (92.3%) Tierärzte teilnahmen. Ein Konsens wurde darüber erzielt, das Havemeyer‐Grading‐System zu übernehmen, die endoskopischen Techniken vor der Auktion zu standardisieren und ein vierstufiges Risikobewertungssystem für YLF einzuführen. Die Grade I und II.1 YLF wurden als risikoarm eingestuft, Grad II.2 als risikoarm bis mäßig, Grad III.1 als mäßig und ≥ Grad III.2 als hoch risikobehaftet. Deskriptoren der Risikokategorisierung auf Grundlage der beobachteten YLF wurden entwickelt.

**Haupteinschränkungen:**

Die Mitglieder des Lenkungsausschusses waren an früheren Forschungsarbeiten beteiligt.

**Schlussfolgerungen:**

Diese Studie bildet die Grundlage für evidenzbasierte Leitlinien, die Transparenz, Konsistenz und den Tierschutz bei der endoskopischen Untersuchung im Rahmen von Jährlingsauktionen verbessern und fundierte Kaufentscheidungen ermöglichen.

## 1 INTRODUCTION

Yearling sales endoscopy is performed at Thoroughbred auctions internationally, to predominantly detect laryngeal dysfunction. Pre‐sale endoscopic guidelines are published by veterinary bodies, while the conditions of sale for post‐sale endoscopy are published by auction houses. Australian and New Zealand protocols are closely aligned due to strong industry ties, whereas yearling endoscopic practices in other countries vary slightly.^1^, ^2^, ^3^, ^4^, ^5^, ^6^, ^7^

In Australasia, yearling laryngeal function (YLF) is graded against auction house criteria during post‐sale examinations.^2^, ^3^ Historically, the 5‐point (Lane) scale was used, with grades 1–3 considered acceptable.^8^ Concerns about the racing potential of yearlings graded 3/5 led to widespread pre‐sale endoscopy, despite limited evidence.^9^, ^10^, ^11^ Horses with grade 3/5 YLF also achieved lower clearance rates and sales prices.^9^ Recent stakeholder engagement revealed low confidence in the Australian endoscopy process, citing unclear links between YLF grade and performance, and poor interobserver agreement due to inconsistent grading practices.^9^

Yearling endoscopic practices in the United Kingdom (UK) and Ireland are very similar and pre‐sale endoscopy is routine. Post‐sale, a horse may be returned if it is heard to make a characteristic abnormal inspiratory noise during active exercise (‘wind test’) and is diagnosed with recurrent laryngeal neuropathy (RLN) on endoscopic examination. No such exercise test is used in Australia, New Zealand or the United States.^7^ The definition of RLN in the UK and Ireland is left open for interpretation by the examiner, while the US auction houses define RLN as consistent immobility or inability to fully abduct the arytenoid cartilage, but no particular grade or grading system is specified.^5^, ^7^

Australian studies examining YLF and future outcomes used an objective adaptation of the Havemeyer grading system.^12^, ^13^, ^14^, ^15^ This adaptation defines the time frame over which full symmetrical arytenoid cartilage abduction must be maintained to be classified as a Havemeyer grade II.2 (≥0.2 s), distinguishing it from grade III.1 (<0.2 s), in which full abduction cannot be maintained.^15^ Yearlings graded ≥II.2 had an increased risk of laryngeal dysfunction requiring surgery, while those graded ≥III.1 were associated with reduced race performance.^12^, ^13^ These findings support broader adoption of the Havemeyer system and evidence‐based guidelines to improve consistency and industry confidence.

A Delphi study was conducted to develop expert veterinary consensus on the Australasian yearling sales endoscopy process. This method uses multiple rounds of anonymous questionnaires with controlled feedback, reducing response bias and overcoming geographical barriers, compared to face‐to‐face discussions.^16^, ^17^ Delphi studies have previously been used to inform guidelines on equine stress indicators, sport horse welfare, colic assessment, and veterinary employability.^18^, ^19^, ^20^, ^21^

Study aims:

- Develop consensus on the most appropriate method for grading YLF.
- Determine the clinical relevance of each YLF grade on race performance and surgical risk.

An additional aim was to develop evidence‐based guidelines to standardise practice and improve industry confidence.

## 2 MATERIALS AND METHODS

### 2.1 Study design

This study built expert veterinary consensus on the Australasian yearling sales endoscopy process using a modified Delphi method. Experts in yearling endoscopy participated in multiple online survey rounds. An anonymised summary of the group's responses was provided to all participants prior to the next survey round to allow them to consider their own responses in light of their peers. Unlike the traditional Delphi studies that start with an open‐ended survey, this study structured the first round around the outcomes of recent yearling endoscopy research to streamline the consensus process and enhance expert engagement.^17^, ^18^, ^19^, ^20^, ^21^, ^22^ This study adhered to the ACCORD reporting guidelines for consensus‐based studies (Table S1).^23^

### 2.2 Steering committee

The steering committee was comprised of four specialist equine veterinarians in equine surgery and/or equine sports medicine and rehabilitation (BHA, BJA, JH, SF). All members had clinical and research experience in yearling endoscopy within Australia and/or New Zealand. One committee member was also an Australian equine veterinary industry body representative, with involvement with the New Zealand equivalent industry body (BHA).

### 2.3 Delphi panel composition and recruitment

Prospective Delphi panellists were identified from equine veterinary body contact lists and chosen by the steering committee (centralised oversight). Panellists had to be actively involved in performing and/or interpreting yearling endoscopic examinations in Australia and/or New Zealand, with a minimum of 10 years of industry experience and deemed to be experts in this field by the steering committee. To minimise bias, none of the steering committee members participated in the Delphi panel. The study aimed to recruit 30–50 panellists, as this was consistent with recommendations for homogenous Delphi studies.^17^ No remuneration was offered for participation.

### 2.4 Development of first Delphi round

The first Delphi round was structured by the steering committee to build on recent literature performed by our research team on the Australasian yearling sales endoscopy process. This research commenced with a stakeholder engagement study that identified areas of industry concern, followed by investigations into the association between yearling laryngeal function and future outcomes.^9^, ^12^, ^13^, ^15^ Piloting of the study materials was not performed outside of the research team. The first Delphi round questionnaire is available as Survey S1.

### 2.5 Delphi method process

Two to four Delphi rounds were planned between late December 2024 and April 2025, with each round delivered via an online survey platform (Qualtrics). Consensus was defined a priori as ≥75% panellist agreement, to reflect a strong majority agreement while minimising survey fatigue.^23^

For the first round, potential panellists were emailed a personalised link to the survey platform, which led to information on the study rationale, relevant literature (see Section 2.4), an embedded consent form, followed by the first‐round questions (Survey S1). Panellists had 3 weeks to complete each survey round, with reminder emails scheduled for non‐responders at 1 week and again 2 days before the deadline.

Each Delphi round consisted of both closed‐ended questions and open‐ended questions. Panellists were given opportunities in each round to provide free‐text comments explaining their reasoning, suggest alterations to the item text, or suggest additional items for subsequent rounds.

After each survey round, the anonymised data were reviewed by the steering committee. Descriptive statistics were used to analyse the closed‐ended questions, with data presented as n (% agreement). Participant responses to the open‐ended questions were coded and thematically analysed using qualitative data analysis software (Nvivo 12 Plus, Lumivero).^24^

Subsequent survey rounds included an anonymised collective summary of the previous round's findings, including both quantitative and qualitative findings. Items that reached consensus were removed from subsequent rounds, to streamline the process and to maintain panellist focus on unresolved items. In instances where the same item did not achieve consensus after two rounds, but there was a strong majority (>65% agreement), the committee opted for a majority ruling.^25^ Completion of each round was required for continued participation; those who did not complete a round were not sent the next survey. New questions were developed in response to panellist feedback. Final determinations for item inclusions in subsequent rounds, including the development of new questions, and the number of rounds held, were made following structured committee discussions, to ensure the questions were aligned with panellist feedback.

## 3 RESULTS

Three Delphi rounds were conducted between late December 2024 and April 2025. A fourth round was deemed unnecessary as consensus, or a majority ruling, was achieved on all items directly related to the study aims by the third round.

The second and third survey questionnaires, developed in response to the previous rounds' responses, are provided as Survey S2 and Survey S3. The collective summary of round 3 data presented to panellists after completion of the study is provided as Table S2.

A total of 63 veterinarians were invited to participate in the study, with 40 veterinarians participating in the first‐round survey (63.5% response rate; Australia *n* = 20; New Zealand *n* = 20). Of these, 39 completed the second round (97.5% response rate; Australia *n* = 20; New Zealand *n* = 19) and 36 out of 39 eligible participants completed the third round (92.3% response rate; Australia *n* = 19; New Zealand *n* = 17).

### 3.1 Round 1

#### 3.1.1 Panellist concerns about the yearling sales endoscopy process

Thematic analysis of panellists' concerns regarding the Australasian yearling sales endoscopy process identified the following main themes:

- Interobserver variability was a prominent concern, with differences in grading of YLF linked to client affiliation and the challenge of interpreting intermediate YLF grades (grade 3 Lane; grade II.2/III.1 Havemeyer).^8^, ^14^ Poor video quality and variability in an individual horse's laryngeal function during the same examination were also cited as contributing factors.
- Variability in pre‐sale endoscopic technique was frequently reported. Governing body pre‐sale endoscopy guidelines were not being consistently followed, and methods such as nasal occlusion, external stimuli, and exercising of horses to alter YLF were discussed, raising questions about the lack of standardisation and horse welfare.^1^
- Intra‐horse variability was raised, with horses reported to display differing degrees of laryngeal function across different timepoints. Multiple examinations and varying techniques were often used to obtain an optimal recording.
- Conflicts of interest were identified, as pre‐sale examinations are often aimed at representing the best possible YLF and veterinarians are not always independent of vendors and buyers. This raised concerns about transparency and consistency of grading.
- Finally, education was emphasised as being essential for all stakeholders, including the limitations of resting endoscopy, the implications of intra‐horse variability, and the correct application of grading systems to ensure fair and informed decision making.

#### 3.1.2 Endoscopic grading system

Among the 40 panellists, the preferred endoscopic grading system selected for evaluating YLF was the 7‐point Havemeyer grading system in 30 panellists (75%, consensus achieved). Eight panellists (20%) selected the 5‐point Lane grading system and two panellists selected neither grading system (5%).

#### 3.1.3 Pass/fail benchmark for YLF grade on a post‐sale endoscopic examination

Panellist selections for the cut‐off between a pass or fail in a yearling post‐sale endoscopic examination in terms of the YLF grade were as follows: 31 panellists (77.5%, consensus achieved) selected that ≤grade III.1 YLF should constitute a pass and grades III.2, III.3 and IV should constitute a fail (as per the current pass/fail benchmark at yearling sales); 5 panellists (12.5%) felt the degree of YLF that constitutes a fail on a post‐sale examination should change, *n* = 5 (12.5%); and 4 panellists (10%) were unsure.^2^

#### 3.1.4 Risk ratings for YLF grades

Panellists were asked if they supported the use of risk rating categories to communicate YLF, like the risk ratings used to describe yearling radiographic findings. Responses were as follows: Yes—30 (75%, consensus achieved); No—4 (10%); and Unsure—6 (15%).

For each YLF grade, panellists selected one of five risk ratings (low‐, low‐moderate‐, moderate‐, moderate‐high‐, or high‐risk) to describe the risk of reduced future performance. Consensus was achieved in round 1 for grades I (low risk), II.1 (low risk), III.3 (high risk), and IV (high risk). Consensus was not achieved in round 1 for grades II.2, III.1, and III.2. The item results from rounds 1 and 2 are displayed in Table 1.

**TABLE 1:** The expert veterinary panellist risk ratings of reduced future performance for each yearling laryngeal function (YLF) grade,^14^ in round 1 (*n* = 40 panellists) and round 2 (*n* = 39) of a Delphi survey into the Australasian yearling sales endoscopy process.

YLF grade	Low risk		Low‐moderate risk		Moderate risk		Moderate‐high risk		High risk		Consensus reached	Agreed risk level
Round	1	2	1	2	1	2	1	2	1	2		
I	40 (100%)	‐	0 (0%)	‐	0 (0%)	‐	0 (0%)	‐	0 (0%)	‐	Yes	Low
II.1	39 (97.5%)	‐	1 (2.5%)	‐	0 (0%)	‐	0 (0%)	‐	0 (0%)	‐	Yes	Low
II.2	14 (35%)	8 (20.5%)	22 (55%)	30 (76.9%)	4 (10%)	1 (2.6%)	0 (0%)	0 (0%)	0 (0%)	0 (0%)	Yes	Low‐moderate
III.1	1 (2.5%)	1 (2.6%)	7 (17.5%)	2 (5.1%)	23 (57.5%)	32 (82.1%)	7 (17.5%)	4 (10.3%)	2 (5%)	0 (0%)	Yes	Moderate
III.2	0 (0%)	0 (0%)	0 (0%)	0 (0%)	7 (17.5%)	2 (5.1%)	14 (35%)	16 (41.0%)	19 (47.5%)	21 (53.8%)	No	Moderate‐high or high^a^
III.3	0 (0%)	‐	0 (0%)	‐	0 (0%)	‐	8 (20%)	‐	32 (80%)	‐	Yes	High
IV	0 (0%)	‐	0 (0%)	‐	0 (0%)	‐	1 (2.5%)	‐	39 (97.5%)	‐	Yes	High

*Note*: Numerical data are presented as *n* (% agreement). Consensus was defined as ≥75% agreement. Items that reached consensus in round 1 were removed from subsequent voting rounds.

^a^ 94.9% of panellists agreed the grade should be either moderate‐high or high risk.

For each YLF grade, panellists selected one of five risk ratings to describe the risk of developing laryngeal dysfunction, requiring surgical intervention. Consensus was achieved in round 1 for grades I (low risk), II.1 (low risk), III.3 (high risk) and IV (high risk), whereas consensus was not achieved in round 1 for grades II.2, III.1 and III.2. The item results from rounds 1 and 2 are displayed in Table 2.

**TABLE 2:** The expert veterinary panellist risk ratings of developing laryngeal dysfunction, requiring a prosthetic laryngoplasty, for each yearling laryngeal function (YLF) grade,^14^ in round 1 (*n* = 40 panellists) and round 2 (*n* = 39) of a Delphi survey into the Australasian yearling sales endoscopy process.

YLF grade	Low risk		Low‐moderate risk		Moderate risk		Moderate‐high risk		High risk		Consensus reached	Agreed risk level
Round	1	2	1	2	1	2	1	2	1	2		
I	39 (97.5%)	‐	1 (2.5%)	‐	0 (0%)	‐	0 (0%)	‐	0 (0%)	‐	Yes	Low
II.1	39 (97.5%)	‐	1 (2.5%)	‐	0 (0%)	‐	0 (0%)	‐	0 (0%)	‐	Yes	Low
II.2	9 (22.5%)	10 (25.6%)	26 (65%)	27 (69.2%)	2 (5.1%)	2 (5.1%)	0 (0%)	0 (0%)	0 (0%)	0 (0%)	No	Low‐moderate^a^
III.1	0 (0%)	0 (0%)	5 (12.5%)	5 (12.8%)	23 (57.5%)	28 (71.8%)	9 (22.5%)	9 (22.5%)	1 (2.5%)	1 (2.5%)	No	Moderate^a^
III.2	1 (2.5%)	0 (0%)	2 (5%)	0 (0%)	5 (12.5%)	4 (10.2%)	13 (32.5%)	8 (20.5%)	19 (47.5%)	27 (69.2%)	No	High^a^
III.3	0 (0%)	‐	0 (0%)	‐	2 (5%)	‐	7 (17.5%)	‐	31 (77.5%)	‐	Yes	High
IV	0 (0%)	‐	0 (0%)	‐	0 (0%)	‐	1 (2.5%)	‐	39 (97.5%)	‐	Yes	High

*Note*: Numerical data are presented as *n* (% agreement). Consensus was defined as ≥75% agreement. Items that reached consensus in round 1 were removed from subsequent rounds.

^a^ Chosen by a majority ruling (>65% agreement), as consensus not reached after two rounds.

Qualitative analysis of panellist reasonings behind their assigned risk ratings is available in Survey S3. Analysis identified there was good support for the use of risk ratings, and panellists assigned their risk ratings based on their own clinical experience and/or the scientific data. However, some participants felt the risk ratings and risk tables required further definition and that additional factors should also be factored in when assigning risk ratings. The presentation of two different types of risk was welcomed by some and questioned by others.

### 3.2 Round 2

#### 3.2.1 Risk ratings for YLF grades

The round 2 risk ratings for reduced future performance are displayed in Table 1. Consensus was achieved for YLF grades II.2 (low‐moderate risk) and grade III.1 (moderate risk). Consensus was not achieved for grade III.2; however, responses remained relatively stable between round 1 and 2; therefore, it was considered unlikely that consensus would be achieved after an additional survey round. A substantial majority (94.9%) of participants selected a moderate‐high or a high‐risk rating of reduced future performance for grade III.2.

The round 2 risk ratings for laryngeal dysfunction requiring a LP are displayed in Table 2. Consensus was not achieved after round 2 for grade II.2, III.1 or III.2, with responses remaining relatively stable. It was considered unlikely that consensus would be achieved after another survey round; therefore, a majority ruling was made (>65% agreement) for each grade as follows: grade II.2 was assigned a low‐moderate risk rating (69.2% agreement); grade III.1 was assigned a moderate risk rating (71.8% agreement); and grade III.2 was assigned a high risk rating (69.2% agreement).

#### 3.2.2 Endoscopic technique and grading of YLF

Due to panellist responses in round 1, additional items were developed regarding endoscopic technique and intra‐horse variability.

Panellists were asked if they supported the use of light exercise prior to pre‐sales endoscopy and responded as follows: Yes—21 (53.8%); No—11 (28.2%); Unsure—7 (17.9%); and Prefer not to say—0 (0%). There was some support for the use of light exercise in yearlings, whereas other panellists felt that the sales environment should provide sufficient stimulation to facilitate a diagnostic examination. Multiple concerns were raised about the use of exercise, including the need for standardisation, the practicality of its implementation, and concerns around horse and handler safety. Due to the heterogeneity of responses, the limited data available to assess the effect of light exercise on YLF, and panellist concerns surrounding its implementation, the item was not presented in subsequent rounds.

Panellists were asked which nostril/nasal passage they used when performing pre‐sales endoscopy, with responses as follows: Left—20 (51.3%); Right—14 (35.9%); Variable—5 (12.8%); and Prefer not to say—0 (0%). When asked whether they use nasal occlusion when assessing YLF during pre‐sales endoscopy, responses were: Variable—20 (51.3%); No—14 (35.9%); Yes—5 (12.8%); and Prefer not to say—0 (0%). These items were excluded from subsequent rounds, as the nostril used and use of nasal occlusion by participants would be unlikely to change during the study. Additionally, there is insufficient data to determine the effect of nasal side and nasal occlusion on YLF, to build evidence‐based recommendations.

Panellists were asked how YLF should be graded if variable degrees of laryngeal function are present during the same endoscopic examination. Their responses were as follows: Graded as the most common YLF grade observed—25 (65.1%); Graded as the best YLF grade observed—10 (25.6%); Graded as the worst YLF grade observed—4 (10.3%); Unsure—0 (0%); and Prefer not to say—0 (0%). Following review by the steering committee, this item was excluded from subsequent rounds because it deviates away from the primary study aims.^23^

Items relating to pre‐sale endoscopic guidelines were developed in response to panellist responses in round 1.^1^ Responses (*n* = 39) are presented in Table 3.

**TABLE 3:** Expert veterinary panellist responses (*n* = 39) in a Delphi study when asked questions related to the thoroughbred yearling pre‐sale endoscopic guidelines provided by their governing body.^1^

Pre‐sale guidelines	Yes	No	Unsure	Prefer not to say	Consensus reached
Do you agree that part of the recording should provide an overview of resting (unstimulated) laryngeal movements for 5–10 s?	32 (82.1%)	5 (12.8%)	2 (5.1%)	0 (0%)	Yes
Do you agree that hitting, jabbing, or frightening a horse to stimulate laryngeal movements is not acceptable on animal welfare grounds at any time?	32 (82.1%)	4 (10.3%)	1 (2.6%)	0 (0%)	Yes
Do you agree that the deliberate use of noise or excessive transendoscopic water spray while recording are not acceptable on animal welfare grounds at any time?	24 (61.5%)	9 (23.1%)	4 (10.3%)	2 (5.1%)	No
Do you agree that a minimum of three distinct swallows should be recorded, which clearly show the degree of abduction of both arytenoid cartilages and allow evaluation of the whole larynx during swallowing?	36 (92.3%)	3 (7.7%)	0 (0%)	0 (0%)	Yes

*Note*: Data are presented as *n* (% agreement). Consensus was defined as ≥75% agreement.

Analysis of panellist responses to the item relating to the deliberate use of transendoscopic water spray and/or noise revealed it lacked specificity and involved two separate questions. Many panellists felt that excessive transendoscopic water spray should be discouraged, but a small amount was deemed necessary to obtain a diagnostic examination. Responses regarding the use of noise concluded that frightening or injuring the horse was never acceptable, but some noises could be useful as a distraction technique to improve safety and horse welfare. A decision was made to remove this item from subsequent rounds, due to its ambiguity and to maintain alignment with the predefined study aims.^23^

### 3.3 Round 3

#### 3.3.1 Use of a single risk rating table

Due to the similarities between the agreed risk ratings developed over the two rounds for the risk of reduced future performance and the risk of developing laryngeal dysfunction, panellists were asked if they supported use of a single risk rating table (Table S2). Responses (*n* = 36) were as follows: Yes—34 (94.4%, consensus achieved); Unsure—1 (2.8%); Prefer not to answer—1 (2.8%); and No, I prefer use of two separate risk tables—0 (0%).

A single risk table was constructed by the steering committee with descriptors of the three yes/no decisions required to categorise YLF into the four risk ratings, to align with panellist round 2 feedback (Table S3).^15^ Four colours (red [High Risk], orange [Moderate Risk], yellow [Low‐moderate Risk] and green [Low Risk]) were used to assist with visual interpretation. Panellist responses on whether they agreed with the use of this table when grading and communicating the risk of YLF were as follows: Yes—31 (86.1%, consensus achieved); Unsure—2 (5.6%); Prefer not to say—2 (5.6%); and No—1 (2.8%). Open‐ended responses revealed substantial support for the use of the proposed single risk rating table. However, there were mixed feelings about its layout. In response to panellist feedback, minor modifications were made to the single risk table, as presented in Table 4.

**TABLE 4:** Agreed risk ratings developed by expert veterinary consensus and descriptors of the three yes/no decisions required to categorise yearling laryngeal function (YLF) into four risk levels.

Risk^a^	YLF^b^	Full arytenoid cartilage abduction is achieved	Full arytenoid cartilage abduction is maintained on average for ≥0.2 s^c^	Arytenoid cartilage movements are symmetrical, or there is transient asynchrony/flutter
Low	I, II.1	Yes	Yes	Yes
Low‐moderate	II.2	Yes	Yes	No
Moderate	III.1	Yes	No	No
High	III.2, III.3, IV	No	No	No

^a^ Risk of developing laryngeal dysfunction that might impair race performance and/or require surgical intervention.

^b^ Havemeyer laryngeal function grading system.^14^

^c^ Definition of maintained abduction.^15^

### 3.4 Final outcomes

Expert consensus on endoscopic technique was achieved on the following statements:

- Part of the recording should provide an overview of resting (unstimulated) laryngeal and pharyngeal movements for 5–10 s, to allow the endoscopist/viewer to assess laryngeal function while unstimulated.
- Hitting, jabbing, or frightening a horse to stimulate laryngeal movements is not acceptable on animal welfare grounds at any time.
- A minimum of three distinct swallows should be recorded, which clearly show the degree of abduction of both arytenoid cartilages and allow evaluation of the whole larynx during swallowing.

Expert consensus on endoscopic interpretation was achieved on the following statements:

- Yearling laryngeal function should be graded using the Havemeyer grading system.^14^
- On a post‐sale endoscopic exam, grades III.2, III.3 and IV should constitute a fail (‘does not meet conditions of sale’) and ≤grade III.1 should constitute a pass (‘meets the conditions of sale’).
- For each YLF grade, a single risk rating can be used to communicate the risk of reduced future performance and/or the risk of developing laryngeal dysfunction requiring a LP.
- Yearling laryngeal function can be categorised into one of four risk ratings: low, low‐moderate, moderate, or high risk (Table 4).

## 4 DISCUSSION

This Delphi study successfully established expert veterinary consensus on key aspects of the Australasian yearling sales endoscopy process, addressing previously raised stakeholder concerns and contributing to the development of professional guidelines to improve industry confidence.^9^ This anonymous, iterative approach used the collective intelligence of expert veterinarians to reevaluate YLF grading in light of recent research, develop a clinical risk rating for YLF grades, and establish best practices for yearling pre‐sale endoscopic examinations.^9^, ^12^, ^13^, ^15^, ^17^, ^23^ The panellists maintained a high level of engagement throughout all three rounds, with the expert panel only reducing in size by 10% by the final round, which is considerably better than the 19% average reported in a scoping review.^26^

Two risk rating tables were initially presented, due to recent studies investigating YLF grades with future outcomes.^12^, ^13^ Horses with grade II.2 YLF were found to not be at increased risk of reduced race performance, compared to grade I and II.1.^13^ However, they did have a higher likelihood of developing laryngeal dysfunction requiring surgery.^12^ In contrast, horses with grade III.1 YLF had an increased risk of both reduced performance and surgical intervention.^12^, ^13^ Despite these differences, panellists generally assigned similar risk levels across both risk rating tables. Consensus on the risk of reduced future performance was reached over two rounds for all YLF grades, except grade III.2. However, it is notable that for grade III.2, an overwhelming majority (94.9% agreement) rated the risk as either moderate‐high or high, indicating strong alignment despite not meeting the predefined consensus threshold. Similarly, consensus on the risk of requiring a LP was achieved over two rounds for four YLF grades (I, II.1, III.3 and IV). For the remaining grades (II.2, III.1 and III.2), the predefined criterion for a majority ruling (>65% agreement) was met after round 2. These items align with the study's predefined methodology, ensuring that the results remain robust and accurately reflect expert opinion. The strong consensus achieved in round 3 (94.4% agreement) supporting the use of a single risk table may offer a pragmatic and clinically applicable tool for sales veterinarians when communicating risk to other stakeholders. Additionally, it aligns with the established risk ratings used to communicate yearling radiographic findings.^27^

The consensus achieved on grading YLF using the Havemeyer system, rather than the previously used 5‐point system, provides clarity within the industry and aligns with existing research findings.^8^, ^12^, ^13^, ^14^ Additionally, this consensus aligns with the recent changes to the conditions of sale listed by all 3 major auction houses in Australasia.^28^ One of the strengths of the Havemeyer grading system is that it offers more granularity in assessing horses with prolonged laryngeal asymmetry, by separating the Lane grade 3 out of 5's into two distinct categories based on the duration full arytenoid abduction is maintained. Due to the aforementioned differences in future outcomes between YLF grades II.2 and III.1, the agreed risk ratings of low‐moderate risk and moderate risk, respectively, are justified.

Interobserver variability in grading of laryngeal function has been reported elsewhere, which adds complexity in a pre‐purchase context where the outcome of a single examination can influence buying decisions.^29^, ^30^, ^31^, ^32^ The use of different grading systems and differing interpretations and/or adaptations of the same grading systems were identified as a source of interobserver variability in an Australian stakeholder engagement study.^9^ Potentially, the adoption of a single evidence‐based grading system could assist in reducing variability in YLF grading internationally. Other factors that might potentially influence laryngeal function grading include intra‐horse variation when examined at different time periods and the use of differing endoscopic techniques; however, these factors require further investigation.

Grades I and II.1 YLF were both assigned a low‐risk rating, reflecting previous findings that no significant difference exists between these grades in terms of future race performance or the likelihood of developing laryngeal dysfunction requiring surgical intervention.^10^, ^12^, ^13^, ^33^ Furthermore, the most frequent observer disagreements have been reported between grade I and II.1, demonstrating that differentiation between them is clinically difficult.^12^, ^13^ An additional complication due to the separation of these two grades is that some yearling purchasers perceive a grade I to be superior to all other grades simply due to its numerical order.^9^ Given the clinical equivalence of these grades, separating them is not justified. Assigning the same risk level is expected to enhance interobserver agreement and improve understanding of their clinical significance.

The study maintained strong methodological integrity by defining consensus a priori, ensuring participation was anonymous, and by incorporating structured feedback loops throughout each round. Items with widely variable responses, unclear interpretations, or insufficient empirical backing were removed to keep the study focused on its core aims. The round 2 responses on endoscopic technique allowed us to document variability among participants in the use of nasal occlusion and nostril selection. However, the authors are not aware of any data demonstrating whether nasal occlusion influences YLF grade, and there is only minimal evidence regarding the impact of nostril choice.^34^ Therefore, the steering committee deemed it inappropriate to make recommendations or pursue additional questions on endoscopic technique without empirical evidence. Ethical considerations also remained central to the expert engagement process, ensuring that the findings were both relevant and applicable to industry practice.

The Australian Thoroughbred industry is the second largest in the world, and each year thousands of horses are exported globally from Australia and New Zealand.^35^, ^36^ While this study focuses on the Australasian yearling sales endoscopy process, the similarity of yearling endoscopic procedures in major markets such as the UK, Ireland, and US suggests these findings may inform international practices.^4^, ^5^, ^6^, ^7^ In addition, the structured Delphi methodology offers a robust framework for achieving consensus and developing evidence‐based guidelines in other areas of equine veterinary practice worldwide.

Despite the strengths of this study, several limitations should be acknowledged. The steering committee's prior involvement in related research introduces a potential source of bias. To mitigate this, committee members refrained from voting in all panel rounds. Delphi studies often draw on experts with domain knowledge to develop the initial framework, as few alternative sources exist for generating relevant, evidence‐based items.^17^ Subsequent rounds focused on refining and validating items through panellist feedback, ensuring that consensus reflected the wider expert group, rather than the researchers. Another potential limitation was that response variability in some areas indicated a need for further clarification or refinement of definitions. In particular, qualitative feedback of the additional questions developed for round 2 regarding aspects of endoscopic technique and intra‐horse variability revealed some participants were unclear about what the question was asking and that some questions asked about two separate issues in the same question. Future research should investigate intra‐horse variability, including the effect of different endoscopic techniques on an individual's YLF. Finally, while expert consensus was achieved on multiple items, the Delphi method essentially reflects the perspectives of the expert panel based in Australia and New Zealand and may not fully capture broader industry views.

## 5 CONCLUSION

This Delphi study identified the Havemeyer grading system to be the preferred method for assessing YLF, and developed a unified, clinically relevant risk rating table to communicate the implications of each YLF grade to yearling industry stakeholders. Additionally, the consensus reached on pre‐sale endoscopic techniques can be used as a foundation for the development of evidence‐based professional guidelines to enhance transparency, improve stakeholder confidence, and promote animal welfare. While some areas warrant further investigation, such as intra‐horse variability and the impact of endoscopic technique, the outcomes of this study represent a significant step toward standardising the yearling sales endoscopy process across Australia and New Zealand.

## FUNDING INFORMATION

Open access publishing facilitated by Adelaide University, as part of the Wiley – Adelaide University agreement via the Council of Australasian University Librarians.

## CONFLICT OF INTEREST STATEMENT

The authors declare no conflicts of interest.

## AUTHOR CONTRIBUTIONS

**Josephine L. Hardwick:** Conceptualization; investigation; writing – original draft; methodology; project administration; formal analysis; data curation; resources; visualization; software; validation. **Benjamin J. Ahern:** Writing – review and editing; supervision; visualization; investigation; conceptualization; methodology. **Brian H. Anderson:** Investigation; writing – review and editing; conceptualization; methodology; supervision. **Samantha H. Franklin:** Investigation; writing – review and editing; supervision; resources; conceptualization; methodology; validation; visualization.

## DATA INTEGRITY STATEMENT

Josephine L. Hardwick had full access to all the data in the study and takes responsibility for the integrity of the data and the accuracy of data analysis.

## ETHICAL ANIMAL RESEARCH

Ethics approval was granted by Adelaide University's Human Research Ethics Committee (approval number: H‐2024‐252).

## INFORMED CONSENT

Explicit informed consent for this study was obtained from all study participants.

## Supporting information


**Data S1:** Survey S1.

**Data S2:** Survey S2.

**Data S3:** Survey S3.

**Data S4:** Survey S4.

**Table S1.** Accurate consensus reporting document (ACCORD) detailing how this Delphi study into the Australasian yearling endoscopy process aligns with the ACCORD reporting guidelines.

**Table S2.** The expert consensus risk categories for yearling laryngeal function (YLF) grades produced in rounds 1 and 2, were tabulated and presented in round 3.^14^ Consensus was defined as ≥75% agreement. ^†^Chosen by a majority ruling (>65% agreement), as consensus not reached after two rounds. ^ǂ^94.9% of panellists agreed the grade should be either moderate‐high or high risk.

**Table S3.** The agreed risk levels developed by expert consensus on the yearling endoscopy process and descriptors of the three key yes/no decisions required to categorise yearling laryngeal function grade^14^ into the four risk levels, as presented to participants in round 3 of the Delphi survey. ^†^Definition of maintained abduction.^15^

## Data Availability

The data that support the findings of this study are available upon reasonable request from the corresponding author. Open data sharing exemption is granted by the editor.
