# Supplementary material for: Delphi consensus on Thoroughbred yearling sales endoscopy in Australasia
Source: Equine Vet J. 2026 Apr 14;58(5):1283–92. doi: 10.1002/evj.70164 (PMC13447881; doi:10.1002/evj.70164)
Supplement: Supplementary file 1 [file EVJ-58-1283-s001.pdf]

Table S3: The agreed risk levels developed by expert consensus on the yearling endoscopy process and descriptors of the three key yes/no decisions required to categorise yearling laryngeal function grade[14] into the 4 risk levels, as presented to participants in round 3 of the Delphi survey. †Definition of maintained abduction.[15]

Risk of developing laryngeal dysfunction that might impair race performance and/or require surgical intervention	Yearling laryngeal function grade	Full arytenoid cartilage abduction is achieved at any time	Full arytenoid cartilage abduction is maintained on average for ≥ 0.2 secs†	All arytenoid cartilage movements are symmetrical, or there is transient asynchrony/flutter
Low	I, II.1	Yes	Yes	Yes
Low-moderate	II.2	Yes	Yes	No
Moderate	III.1	Yes	No	No
High	III.2, III.3, IV	No	No	No
