# Supplementary material for: Delphi consensus on Thoroughbred yearling sales endoscopy in Australasia
Source: Equine Vet J. 2026 Apr 14;58(5):1283–92. doi: 10.1002/evj.70164 (PMC13447881; doi:10.1002/evj.70164)
Supplement: Supplementary file 2 [file EVJ-58-1283-s002.pdf]

Survey S2

Default Question Block

A Delphi study to formulate expert consensus on the yearling sales endoscopy process: Round 2

Thank you for your responses to the round 1 questionnaire. We were pleased with the response rate and level of engagement from invited participants, and we have already achieved consensus on several points.

In round 2, we present an anonymised collective summary of round 1 responses, to give you individual insights on the group trends:

- The responses to the open-ended questions (questions 1, 6 & 8) were coded and analysed thematically via an iterative process, using qualitative data analysis software (Nvivo 12). Major themes were identified and presented in the results below. These themes were used to structure the round 2 questionnaire.
- The responses to the closed-ended questions (questions 2, 3, 4, 5 and 7) were analysed descriptively and the results are presented as a percentage agreement. As outlined in the participant information sheet, for consensus to be achieved on each question ≥ 75% participants had to agree on the answer. Questions for which consensus was achieved in round 1 have been removed from the round 2 questionnaire.

The aim of this Delphi study is to develop an expert consensus on multiple points relating to yearling endoscopy. The collective summary allows you to consider the responses of your peers, prior to re-answering the questions that did not achieve consensus in round 1.

Several new questions have also been added to survey 2, relating to main themes identified

from the open-ended questions answered in round 1.

○ Please click to continue to the round 1 collective summary

Question 1: What are your main concerns about the current yearling sales endoscopy process?

Main themes identified from question 1 responses:

1. Interobserver (inter-vet) variability when grading the same endoscopic examination

- **Participants felt that variability could be dependent on the client**

  Participant 1: "Differences between vendor expectations from lenient grading by own vet and grading at repository or after sale."
  Participant 24: "Veterinarians conducting the examinations are not always fully independent of the vendor or buyer."

- **Yearlings with intermediate laryngeal function are reportedly difficult to grade.**

  Participant 11: "Variation in interpretation of Grade 3 (Lane) between vets."
  Participant 35: "There is a subtlety around the grade 3 (Lane) that in many cases will perform and not deteriorate over time that is difficult to communicate."
  Participant 32: "The differences in opinion particularly regarding scopes that have been graded as a 3 (Lane)."

- **The effect of "poor quality endoscopic videos (participant 9)" on interobserver variability**

Participant 5: "….often associated with the behaviour of the yearling and particularly head movements, total pharyngeal behaviour which can be particularly variable to the point grading from variable sections can be quite different."

2. Variability in endoscopic technique

- **EVA pre- sale endoscopy guidelines are not always adhered to.**

Participant 2: "Many videos are edited for the best 30 seconds of footage (ie the EVA protocols are not being followed at all)."

Participant 23: "The variable quality of the videos, many are not diagnostic in that they vary from the Equine Veterinarians Australia guidelines."

- **Variety of methods used during endoscopy process (also see 'Intra-horse variability')**
- **Exercising prior to exam.**
- **Frequent/continuous laryngeal stimulation.**
- **Nasal occlusion.**
- **The use of external stimuli "so that they are stressed and potential breathe with more exertion."**
- **"Safety" concerns and horses "frightened with noise and visual stimuli (plastic bags)."**

Participant 40: "Not all pre-sale scopes are performed in the same manner eg lunged, medicated."

Participant 11: "Need to standardize methods for acquiring pre-sale endoscopic videos, as it possible to improve most horses a grade from their normal resting function by various methods of stimulation."

3. Intrahorse variability (individual horses displaying different degrees of laryngeal function)

- **How to grade variable laryngeal function during the same examination:**

  Participant 2: “What part of the examination forms the basis of the assessment? The worst part, the best part, or the average? I think that needs clarification.”

- **Multiple exams often performed to achieve optimal recording:**

  Participant 27: “Variability is greatly enhanced when horses are being examined multiple times or situations until the "best" scope is achieved; making people dubious that for horse may be a grade higher in the majority of cases but not shown.”

- **Several variations in endoscopic technique are reportedly used to try and improve laryngeal function grade.**

  Participant 40: “Different operators have different (scoping) skill sets therefore may be able to produce a video that portrays the horse’s laryngeal function in a different (more or less favourable) manner than another endoscopist.”

- **Questions were raised as to if any research had investigated intra-horse variability**

  Participant 36: “Some scopes are clearly obtained with respiratory stimulation - lunging beforehand, nasal occlusion etc etc. Is there any evidence to suggest this can alter the assessment of laryngeal grade and how should these horses be assessed.”

4. Conflicts of interest

- **Pre-sale examinations are highlighting the best possible laryngeal function**
- **May not be independent of the vendor or buyer**

- **Intra-observer variability: difference in grade communicated to vendor Vs purchaser**

  Participant 6: “Veterinarians conducting the examinations are not always fully independent of the vendor or buyer.”

  Participant 8: “Different interpretations between veterinarians on the grade of the same larynx which is disclosed to vendors and buyers.”

5. Education of all stakeholders recommended on the following:

- **The limitation of a single resting endoscopic examination**
- **Variability is not necessarily due to inconsistency between vets.**
- **Changing of the grading system.**

  Participant 21: “The limitations of a standing at rest video scope to fairly assess the functionality of the equine larynx.”

  Participant 40: “where there is a lack of awareness, an overabundance of caution can be found leading to negative impacts on sales.”

  Participant 39: “Any changes to the grading system - pre or post sale - must be implemented with care and ample education of all stakeholders in order to ensure that scopes are correctly graded so that informed decisions can be made that are fair to the horse as well as the buyers, sellers and other stakeholders.”

Researcher response: Considering these themes, several additional questions have been developed and inserted into the round 2 survey regarding intra-horse variability, endoscopic technique and the EVA pre-sale endoscopic guidelines.

○ Please click to continue to question 2 responses

Question 2: Considering recent findings (Hardwick et al 2024b, Hardwick et al 2024c), which grading system do you think should be used to evaluate yearling laryngeal function?

Question 2 responses:

Forty (40) participant responses received:

- Lane grading system (5-point system): **8 participants (20%)**
- Havemeyer grading system (7-point system): **30 participants (75%)**
- Neither: **2 participants (5%)**

Outcome: CONSENSUS ACHIEVED, as >= 75% agreement.

Expert consensus: The Havemeyer grading system is the preferred grading system to evaluate yearling laryngeal function.

○ Please click to continue

Question 3: For post-sale endoscopy, do you agree that yearling laryngeal function ≤ grades III.1 should constitute a pass (meet the conditions of sale) and ≥ grades III.2 and above should constitute a fail (do not meet the conditions of sale)?

Question 3 responses:

Forty (40) participant responses:

- Yes, I agree that on a post-sale endoscopic exam, grades III.2 and III.3 should constitute a fail and ≤ grade III.1 should constitute a pass: **31 participants (77.5%)**
- No, I believe the degree of laryngeal function that constitutes a fail on a post-sale examination should change: **5 participants(12.5%)**
- Unsure: **4 participants (10%)**

Outcome: CONSENSUS ACHIEVED, as >= 75% agreement.

Expert consensus: On a post-sale endoscopic exam, grades III.2 and III.3 should constitute a fail and ≤ grade III.1 should constitute a pass.

○ Click to continue to next question

Question 4: Do you support the use of risk rating categories to evaluate yearling laryngeal function?

Question 4 responses:

Forty (40) participant responses received:

- Yes: **30 participants (75%)**
- No: **4 participants (10%)**
- Unsure: **6 participants (15%)**

Outcome: CONSENSUS ACHIEVED, as >= 75% agreement.

Expert consensus: The use of risk rating categories for yearling laryngeal function are recommended.

Support for the use of risk ratings was also expressed in the written responses:

> Participant 6: "I certainly believe a standardised system would assist with the integrity of both vets and the Australian yearling sales."

◯ Click to continue to the next question

Question 5: Risk rating category: Risk of reduced future performance

For each yearling laryngeal function grade, please select your preferred risk rating category.

Question 5 responses:

Yearling laryngeal function grade	Low risk	Low-moderate risk	Moderate risk	Moderate-high risk	High risk	Consensus achieved?	Agreed risk level
**I**	40 (100%)	0 (0%)	0 (0%)	0 (0%)	0 (0%)	**Yes**	**Low**
**II.1**	39 (97.5%)	1 (2.5%)	0 (0%)	0 (0%)	0 (0%)	**Yes**	**Low**
**II.2**	14 (35%)	22 (55%)	4 (10%)	0 (0%)	0 (0%)	**No**	**-**
**III.1**	1 (2.5%)	7 (17.5%)	23 (57.5%)	7 (17.5%)	2 (5%)	**No**	**-**
**III.2**	0 (0%)	0 (0%)	7 (17.5%)	14 (35%)	19 (47.5%)	**No**	**-**
**III.3**	0 (0%)	0 (0%)	0 (0%)	8 (20%)	32 (80%)	**Yes**	**High**
**IV**	0 (0%)	0 (0%)	0 (0%)	1 (2.5%)	39 (97.5%)	**Yes**	**High**

Table 1: Assigned risk rating of reduced future performance for each laryngeal function grade, from a panel of 40 equine veterinarians.
Data are presented as n (% agreement). For consensus to be achieved, ≥ 75% agreement required.

- **Expert consensus was achieved for grades I (low risk), II.1 (low risk), III.3 (high risk) and IV (high risk).**
- **Expert consensus was not achieved for grades II.2, III.1 and III.2.**

Please consider the group's responses and re-answer the question below.

Question: For yearling laryngeal function grades II.2, III.1 and III.2, please select your preferred category for risk of reduced future performance.

	Low risk	Low-moderate risk	Moderate risk	Moderate-high risk	High risk
**Grade II.2**	◯	◯	◯	◯	◯
**Grade III.1**	◯	◯	◯	◯	◯
**Grade III.2**	◯	◯	◯	◯	◯

Question 6: Please explain your reasoning for the risk ratings given for each laryngeal function grade.

Question 6 responses have been combined with question 8 (see below).

◯ Click to continue

Question 7: Risk rating category: <u>Risk of developing clinical recurrent laryngeal neuropathy (requiring a prosthetic laryngoplasty).</u>

For each yearling laryngeal function grade, please select your preferred risk rating category.

Question 7 responses:

Yearling laryngeal function grade	Low risk	Low-moderate risk	Moderate risk	Moderate-high risk	High risk	Consensus achieved?	Agreed risk level
I	39 (97.5%)	1 (2.5%)	0 (0%)	0 (0%)	0 (0%)	Yes	Low
II.1	39 (97.5%)	1 (2.5%)	0 (0%)	0 (0%)	0 (0%)	Yes	Low
II.2	9 (22.5%)	26 (65%)	5 (12.5%)	0 (0%)	0 (0%)	No	-
III.1	2 (5%)	5 (12.5%)	23 (57.5%)	9 (22.5%)	1 (2.5%)	No	-
III.2	1 (2.5%)	2 (5%)	5 (12.5%)	13 (32.5%)	19 (47.5%)	No	-
III.3	0 (0%)	0 (0%)	2 (5%)	7 (17.5%)	31 (77.5%)	Yes	High
IV	0 (0%)	0 (0%)	0 (0%)	1 (2.5%)	39 (97.5%)	Yes	High

Table 2: Assigned risk rating of developing clinical recurrent laryngeal neuropathy (requiring a prosthetic laryngoplasty) for each yearling laryngeal function grade, from a panel of 40 equine veterinarians.
Data are presented as n (% agreement). For consensus to be achieved, ≥ 75% agreement required.

- **Expert consensus was achieved for grades I (low risk), II.1 (low risk), III.3 (high risk) and IV (high risk).**
- **Expert consensus was not achieved for grades II.2, III.1 and III.2.**

Please consider the group's responses and re-answer the question below.

For yearling laryngeal function grades II.2, III.1 and III.2, please select your preferred category for risk of developing RLN, requiring a prosthetic laryngoplasty.

	Low risk	Low-moderate risk	Moderate risk	Moderate-high risk	High risk
Grade II.2	○	○	○	○	○
Grade III.1	○	○	○	○	○
Grade III.2	○	○	○	○	○

Question 6 and 8 combined: Please explain your reasoning for the risk ratings given for each laryngeal function grade, in terms of risk of reduced future performance and risk of developing clinical RLN (requiring a prosthetic laryngoplasty).

The main themes identified across both question 6 and 8 were:

1. There was good support for the use of risk ratings, supporting the consensus achieved (77.5% agreement) for the use of risk ratings.

Participant 1: *"I would be happy to communicate this with clients.*

2. Participants felt the scientific data reflected their own clinical experience and assigned their risk ratings based on the scientific literature and/or their own experience.

Participant 21: *"Clinical impressions reflect the trends outlined in the statistical breakdown."*

Participant 38: *"Based on the data."*
Participant 10: *"Clinical experience following horses throughout their career from yearlings and then into their race career has imparted my association of risk to each grade."*
Participant 14: *"Based on recent literature and personal experience."*
Participant 28: "*My interpretation of recent research and clinical experience from case follow up.*

3. Discussion around the structure and definition of the risk ratings and risk tables,
"How we define risk is important (participant 36)."

- **There was some support for reducing the number of risk levels from 5 (mild, mild-moderate, moderate, moderate-high, and high-risk) down to 3 (mild, moderate, and high-risk), whereas others supported the 5 risk levels.**

Participant 17: *"There are too many risk categories when including low-moderate; moderate-high. I believe we should use evidence-based medicine to identify horses into one of three categories only. i.e. <20% risk of requiring laryngoplasty = low risk; >20% risk of requiring laryngoplasty/ reduced earnings = moderate risk; >40% risk of requiring laryngoplasty/ significant reduced earnings = high risk."*
Participant 24: *"...having the 5 levels of risk is the right way to go."*

- **The presentation of two different types of risk (risk of reduced future performance and risk of requiring a prosthetic laryngoplasty) was welcomed by some, and questioned by others, particularly by participants that assigned identical grades to both tables.**

Participant 24: *"I think when adapting a risk rating using a two-fold system will be important."*
Participant 35: *"It is unlikely I will be separating my scope reading results into more than one category as the clients can only deal with so much information."*
Participant 36: "Risk to race or risk to need for a tie back. People don't want to buy horses that need a tie-back. The effect on performance is less of an issue in buyers' minds - they don't want a horse that needs a tie-back."

- **Some participants felt that additional factors (i.e. sex, phenotype, intended race age and distance) should also be factored in when assigning risk ratings:**

Participant 5: *"If the purchaser is buying a yearling that will possibly be at elite performance as a 4yo then I would give a high risk versus a yearling that is an early 2yo I would be far more lenient."*
Participant 3: *"A grade 3.1 horse that is male, is big, has easily palpated muscular process is likely to have LLH, however a small filly the has good muscular cover of the muscular process is unlikely to have LLH."*Participant 35: *"It is also highly dependent on the reason for the purchase as yearling bought for resale as a ready to runner 2yo or to sell at a trial would have a very different risk rating compared to those bought for purely racing purposes."*

Researcher response
We created two risk tables (risk of reduced future performance and risk of requiring a prosthetic laryngoplasty), as no difference in future race performance was identified between yearlings with grades I/II.1 and grades II.2 laryngeal function, but there was a significant difference in risk of requiring a prosthetic between grades I/II.1 and II.2.[1,2]
At present, the consensus in risk achieved for grades I, II.1, III.3 and IV is the same for both risk of reduced future performance and risk of requiring a prosthetic laryngoplasty. If this trend continues in round 2, simplifying the risk rating down to just one risk table for each yearling laryngeal function grade could be considered.

In response to comments about consideration of additional factors when assigning risk to yearling laryngeal function, we would like to state the following:

- The risk ratings were exclusively developed to assign a risk to yearling laryngeal function grade alone, to align with robust scientific data.[1,2]
- We envisage the veterinarian, and client, using these risk ratings to formulate their own opinion on the whether that yearling is suitable for purchase, along with all the other risks associated with racehorse ownership (i.e. the risk of radiographic findings).
- A major reason for developing an expert consensus on the risk of different yearling laryngeal function grades, is to improve consistency between veterinarians and address stakeholder concerns. Formulating an expert consensus on these risk categories should

improve clarity and in turn improve industry confidence in the yearling endoscopy process.

1. Hardwick JL, Ahern BJ, Crawford KL, Allen KJ, Anderson BH, Rose KJ, et al. Yearling laryngeal function grades II.2 and below are not associated with reduced performance. *Equine Vet J*. 2025. https://doi.org/10.1111/evj.14452

2. Hardwick JL, Ahern BJ, Crawford KL, Allen KJ, Franklin SH. Yearling laryngeal function in Thoroughbreds that underwent a laryngoplasty differs from controls. *Equine Vet J*. 2025; 57(2): 431–440. https://doi.org/10.1111/evj.14110

Please feel free to write any comments in the text box below.

Questions 9, 10, 11, 12 and 13 relate to pre-sales endoscopic technique, following analysis of the responses to the open-ended questions in survey 1.

The current 'Guidelines for Pre-Sale Videoendoscopy of the Laryngopharynx of Thoroughbred Horses Prior to Sale in Australia and New Zealand' can be accessed here: Presale guidelines

Question 9: Do you support the use of light exercise prior to a pre-sales endoscopic examination?

- ◯ Yes
- ◯ No
- ◯ Unsure
- ◯ Prefer not to say

Question 10: As per the pre-sale guidelines, do you agree that part of the recording image should provide an overview of resting (unstimulated) laryngeal and pharyngeal movements for 5-10 seconds, to allow the endoscopist/viewer to assess laryngeal function while unstimulated?

(This does not include the judicious use of the air and water channels to provide a clear picture where necessary)

- ◯ Yes
- ◯ No
- ◯ Unsure
- ◯ Prefer not to say

Question 11: As per the guidelines, do you agree that hitting, jabbing, or frightening a horse in an attempt to stimulate laryngeal movements is not acceptable on animal welfare grounds at any time?

- ◯ Yes
- ◯ No
- ◯ Unsure
- ◯ Prefer not to say

Question 12: As per the guidelines, do you agree that the deliberate use of noise or excessive water spray while recording are not acceptable on animal welfare grounds at any time?

- ◯ Yes
- ◯ No
- ◯ Unsure
- ◯ Prefer not to say

Question 13: As per the guidelines, do you agree that a minimum of 3 distinct swallows should be recorded, which clearly show the degree of abduction of both arytenoid cartilages and allow evaluation of the whole larynx during swallowing?

- ◯ Yes
- ◯ No
- ◯ Unsure
- ◯ Prefer not to say

Please feel free to explain your reasoning behind you answers to questions 9-13.

Question 14: Which nostril/nasal passage do you use when performing pre-sales endoscopy?

- ◯ Right side
- ◯ Left-side
- ◯ Variable
- ◯ Prefer not to say

Question 15: Do you use nasal occlusion when assessing laryngeal function during pre-sale endoscopy?

- ◯ Yes
- ◯ No
- ◯ Variable
- ◯ Prefer not to say

Please feel free to write any comments explaining your reasoning

Question 16: How should laryngeal function be graded if a horse shows variable degrees of laryngeal function during the same endoscopic examination?

- ◯ Graded as the most common (average) laryngeal function grade observed
- ◯ Graded as the best laryngeal function grade observed

◯ Graded as the worst laryngeal function grade observed
◯ Unsure
◯ Prefer not to say

Please feel free to write any comments explaining your reasoning

Question 17 (for vets working in Australia): In Australia, the pre-sale endoscopic examination should be performed on the sales complex within 7 or fewer days prior to the commencement of the sale. In New Zealand, the examination may be performed prior to arrival at the sales complex provided it is performed within 7 or fewer days before commencement of the sale.

Question: Do you support a change in Australian pre-sales endoscopy protocol, to allow the examination to be performed prior to arrival at the sales complex, provided that it is still performed within 7 or fewer days before commencement of the sale?

◯ Yes
◯ No
◯ Unsure
◯ Not applicable (NZ vets)/Prefer not to say

Please feel free to write any comments explaining your reasoning

Thank you for completing survey 2.

Please write any other comments and/or justifications for your answers in the space provided.

Powered by Qualtrics
