# Supplementary material for: Delphi consensus on Thoroughbred yearling sales endoscopy in Australasia
Source: Equine Vet J. 2026 Apr 14;58(5):1283–92. doi: 10.1002/evj.70164 (PMC13447881; doi:10.1002/evj.70164)
Supplement: Supplementary file 3 [file EVJ-58-1283-s003.pdf]

Equine Veterinary Journal BEVA

Table S2: The expert consensus risk categories for yearling laryngeal function (YLF) grades produced in round 1 and 2, were tabulated and presented in round 3.[14] Consensus was defined as ≥ 75% agreement. †Chosen by a majority ruling (> 65% agreement), as consensus not reached after 2 rounds. ‡94.9% of panellists agreed the grade should be either moderate-high or high risk.

YLF grade	Risk of reduced future performance	Risk of clinical disease requiring a LP	Overall risk
I	Low	Low	Low
II.1	Low	Low	Low
II.2	Low-moderate	Low-moderate†	Low-moderate
III.1	Moderate	Moderate†	Moderate
III.2	Moderate-high / High‡	High†	High
III.3	High	High	High
IV	High	High	High
