# Supplementary material for: Delphi consensus on Thoroughbred yearling sales endoscopy in Australasia
Source: Equine Vet J. 2026 Apr 14;58(5):1283–92. doi: 10.1002/evj.70164 (PMC13447881; doi:10.1002/evj.70164)
Supplement: Supplementary file 5 [file EVJ-58-1283-s005.pdf]

Survey S3

Default Question Block

A Delphi study to formulate expert consensus on the yearling sales endoscopy process: Round 3 (Final round)

Thank you for your responses to the round 2 questionnaire. The level of engagement from participants remained high and further consensus was achieved. Thirty-nine out of 40 participants completed the second survey round (97.5% response rate).

In the 3rd and final round, we present the collective summary of round 2 responses. Additionally, based on the data from this study, we propose the use of a single risk rating table (overall risk) for evaluation of yearling laryngeal function.

In this final round, we are seeking your feedback on the risk rating table and the proposed wording to ensure effective communication with vendors and buyers of Thoroughbred yearlings.

○ Please click to continue

Question 5: Risk rating category: Risk of reduced future performance.

Round 2 question 5 responses (39 participants):

Yearling laryngeal function grade	Low risk	Low-moderate risk	Moderate risk	Moderate-high risk	High risk	Consensus (≥ 75% agreement)	Agreed risk level
II.2	8 (20.5%)	30 (76.9%)	1 (2.6%)	0 (0%)	0 (0%)	Yes	Low-moderate
III.1	1 (2.6%)	2 (5.1%)	32 (82.1%)	4 (10.3%)	0 (0%)	Yes	Moderate
III.2	0 (0%)	0 (0%)	2 (5.1%)	16 (41.0%)	21 (53.8%)	No	Moderate -high / high

Table 1: Assigned risk rating of reduced future performance for each yearling laryngeal function grade, from the 2nd Delphi round of 39 equine veterinarians.

Data are presented as n (% agreement).

OUTCOME:

- **Consensus achieved** between participants on the risk of reduced future performance for **grades II.2 (low-moderate risk)** and **grade III.1 (moderate risk).**
- **Consensus was not achieved for grade III.2** after the 2 survey rounds. Responses remained relatively stable between round 1 and 2, therefore it is unlikely consensus would be achieved after an additional survey round. A **substantial majority (94.9%)** of participants selected a **moderate-high or a high risk rating** of reduced future performance for **grade III.2.**

○ Click to continue to next question.

Question 7: Risk rating category: Risk of developing clinical recurrent laryngeal neuropathy (requiring a prosthetic laryngoplasty).

Round 2 question 7 responses (39 participants):

Yearling laryngeal function grade	Low risk	Low-moderate risk	Moderate risk	Moderate-high risk	High risk	Consensus reached (≥ 75% agreement)	Agreed risk level
II.2	10 (25.6%)	27 (69.2%)	2 (5.1%)	0 (0%)	0 (0%)	No	Low-moderate*
III.1	0 (0%)	5 (12.8%)	28 (71.8%)	9 (22.5%)	1 (2.5%)	No	Moderate*
III.2	0 (0%)	0 (0%)	4 (10.2%)	8 (20.5%)	27 (69.2%)	No	High*

Table 2: Assigned risk rating of developing clinical disease requiring a prosthetic laryngoplasty, for each yearling laryngeal function grade, from the 2nd Delphi round of 39 equine veterinarians.

Data are presented as n (% agreement). *Selected by the majority of participants, as consensus (≥75%) was not achieved after 2 rounds.

OUTCOME:

- **Consensus (≥ 75% agreement) was not achieved for grade II.2, however a majority (69.2%) agreed on a low-moderate risk level.** In survey 1, low-moderate risk was also selected by the majority of participants and participant responses have remained relatively stable. It is unlikely that an additional survey round will change the outcome, therefore we deem it appropriate to assign grade II.2 as low-moderate risk for developing clinical disease, requiring a prosthetic laryngoplasty.
- **Consensus was not achieved for grade III.1, however a majority (71.8%) agreed on a moderate risk level,** therefore for the reasons mentioned above, grade III.1 should be assigned a moderate risk level of developing clinical disease.
- **Consensus was not achieved for grade III.2, however again a majority (69.2%) agreed on a high risk level,** therefore we conclude grade III.2 should be assigned a high risk level of developing clinical disease.

○ Click to continue to next question

The agreed risk ratings for each yearling laryngeal function grade were almost identical for both the risk of reduced future performance and the risk of developing clinical disease, requiring a prosthetic laryngoplasty. Therefore, we propose use of a **single risk table** for each yearling laryngeal function grade, rather than two separate risk tables. We believe that providing a single risk table **simplifies communication** and delivers a **clearer message** to other stakeholders.

The use of a single risk table was also supported by participant responses in both survey 1 and survey 2:

- Veterinarian 11: *"If we could have one risk table that would be ideal as clients are easily confused."*

- Veterinarian 35: *"I will likely continue to provide only 1 risk rating for clients, as to them, reduced performance or requiring a tie-back is one and the same, even if there is some difference in their probabilities."*

Table 3 below shows the agreed risk ratings for both the risk of reduced future performance and the risk of developing clinical recurrent laryngeal neuropathy requiring surgical intervention. The proposed "overall risk" is listed in the final column.

Note: A high overall risk rating assigned to grade III.2, as the majority of participants selected this rating in both risk tables.

Yearling grade[a]	Risk of reduced future performance	Risk of clinical disease requiring a LP	Overall risk
I	Low	Low	**Low**
II.1	Low	Low	**Low**
II.2	Low-moderate	Low-moderate*	**Low-moderate**
III.1	Moderate	Moderate*	**Moderate**
III.2	Moderate-high / High‡	High*	**High**
III.3	High	High	**High**
IV	High	High	**High**

Table 3: The expert consensus risk category for both reduced future performance and developing clinical disease requiring a prosthetic laryngoplasty, was combined to produce an overall risk category for each grade.

[a]Havemeyer laryngeal function grading system (Dixon et al 2003).
*Participant majority used, as consensus (≥75% agreement) not achieved.
‡94.9% agreement of either moderate-high or high risk.

Question: Considering the outcomes of this Delphi survey, do you support the use of a single risk table when evaluating yearling laryngeal function?

- ◯ Yes, I support the use of a single risk table.
- ◯ No, I prefer the use of two separate risk tables.
- ◯ Unsure.
- ◯ Prefer not to answer.

Providing there is participant support for use of a single risk table (previous question), we believe the graphic below (table 4) could be a useful tool for equine veterinarians to refer to when grading and communicating yearling laryngeal function. This graphic **combines the outcomes of this Delphi study**, with a **simplified version of the diagnostic decision tree**

modified specifically for use in yearlings.[1]

Table 4 includes descriptors of the **three key yes/no decisions** required to categorise yearling laryngeal function into the 4 risk levels (low-, low-moderate-, moderate-, or high-risk categories):

1. Is full arytenoid cartilage abduction achieved at any time? (Yes/No)
2. Is full arytenoid cartilage abduction maintained on average for 0.2 seconds or more, following swallowing? (Yes/No)
3. Are all arytenoid cartilage movements symmetrical, or (if present) only transient asynchronous movement or flutter is observed? (Yes/No).

Risk of developing laryngeal dysfunction that might impair race performance and/or require surgical intervention	Yearling laryngeal function grades[a]	Full arytenoid cartilage abduction is achieved at any time	Full arytenoid cartilage abduction is maintained on average for ≥ 0.2 secs[+]	All arytenoid cartilage movements are symmetrical, or there is transient asynchrony/ flutter
Low	I, II.1	Yes	Yes	Yes
Low-moderate	II.2	Yes	Yes	No
Moderate	III.1	Yes	No	No
High	III.2, III.3, IV	No	No	No

Table 4: Agreed risk levels developed by expert consensus and descriptors of the three key yes/no decisions required to categorise yearling laryngeal function into the 4 risk levels.
[a]Havemeyer laryngeal function grading system (Dixon et al 2003).[2]
[+]Definition of maintained abduction (Hardwick at al 2024).[1]

Analysis of participant comments also showed support for inclusion of a description of the degree in laryngeal function that each risk level pertains to.

- **Veterinarian 7**: *"It would be very beneficial if we could have a consensus on the wording of those risk ratings in specific relation to endoscopic examination."*

References

1. Hardwick, J., Ahern, B. and Franklin, S. (2024) A first step towards objective grading of equine laryngeal function. *Vet J. 308, 106259*. https://doi.org/10.1016/j.tvjl.2024.106259
2. Dixon, P., Robinson, E. and Wade, J.F. (2003) Consensus statements on equine recurrent laryngeal neuropathy. *Havemeyer Foundation Monograph Series 11*. https://havemeyerfoundation.org/wp-content/uploads/2021/09/monograph11-Stratford-Monograph.pdf

Question: Do you agree with the risk table presented (table 4) for use when grading and communicating the risk of yearling laryngeal function?

- ◯ Yes.
- ◯ No.
- ◯ Unsure.
- ◯ Prefer not to answer.

Kindly provide any comments on the risk table presented in the space below.

If you disagree with the proposed wording, please suggest alternative options.

Questions 9: Do you support the use of light exercise prior to a pre-sales endoscopic examination?

Question 9 round 2 responses (n = 39):

- **Yes: 21 (53.8%)**
- **No: 11 (28.2%)**

- **Unsure: (17.9%)**
- **Prefer not to say: 0 (0%)**

OUTCOME: Consensus not achieved.

Qualitative analysis of participants reasonings for their response, included some support for light exercise in yearlings, particularly those that are difficult to stimulate during the examination:

- Veterinarian 39: *“Some yearlings come in for scoping in a particularly 'sleepy' state so it is fair that they are 'woken up' with judicious use of light exercise.*”
- Veterinarian 30: “*Light exercise presents the horse in the best possible light and is also closer to what the larynx will behave like in exercise situations*.”

Whereas other participants felt that the yearling should be significantly stimulated from just walking down to the stalls:

- Veterinarian 2: “*The walking down from the stalls to the scoping crush and into a new environment is typically significant stimulation. This is the stimulation that horses received in your post-sale video's utilised in your studies*….”

Multiple concerns were raised by participants, including the need for it to be standardised, as “*light exercise is difficult to define. The yearling should be alert and breathing normally* (veterinarian 19)”. Additionally, “*the practicality of its implementation* (veterinarian 18)” in a sales environment was questioned. Safety concerns were also raised, as in some cases “*there is no safe area to exercise horse or horse not safe to exercise* (veterinarian 40)”.

Researcher response: Ultimately, there is no data available to define the use of light exercise,

or to show the effect of light exercise on yearling laryngeal function, therefore no recommendations can be made at present.

◯ Please click to continue to next question

Question 10: As per the pre-sale guidelines, do you agree that part of the recording image should provide an overview of resting (unstimulated) laryngeal and pharyngeal movements for 5-10 seconds, to allow the endoscopist/viewer to assess laryngeal function while unstimulated?

(This does not include the judicious use of the air and water channels to provide a clear picture where necessary).

Participant responses (n = 39):

Yes: 32 (82.1%)

No: 5 (12.8%)
Unsure: 2 (5.1%)
Prefer not to say: 0 (0%)

OUTCOME:
Consensus achieved. Part of the recording should provide an overview of resting (unstimulated) laryngeal and pharyngeal movements for 5-10 seconds, to allow the endoscopist/viewer to assess laryngeal function while unstimulated.

◯ Please click to continue to the next question

Question 11: As per the guidelines, do you agree that hitting, jabbing, or frightening a horse in an attempt to stimulate laryngeal movements is not acceptable on animal

welfare grounds at any time?

Participant responses (n = 39):

- **Yes: 32 (82.1%)**
- **No: 4 (10.3%)**
- **Unsure: 1 (2.6%)**
- **Prefer not to say: 2 (5.1%)**

OUTCOME: Consensus achieved. Hitting, jabbing, or frightening a horse to stimulate laryngeal movements is not acceptable on animal welfare grounds at any time.

○ Please click to continue to the next question

Question 12: As per the guidelines, do you agree that the deliberate use of noise or excessive water spray while recording are not acceptable on animal welfare grounds at any time?

Participant responses (n = 39):

- **Yes: 24 (61.5%)**
- **No: 9 (23.1%)**
- **Unsure: 4 (10.3%)**
- **Prefer not to say: 2 (5.1%)**

OUTCOME: Consensus not achieved.

Analysis of participant comments related to this question revealed mixed feelings and a need for further clarification and definition of the question being asked.

Many participants felt that excessive use of water spray, particularly down the rima glottidis, should be discouraged. However, participants also highlighted that water spray was vital for laryngeal stimulation, in order to obtain a diagnostic examination.

- Veterinarian 11: "*The constant jetting of a throat to maintain abduction and not allow assessment of symmetry when relaxed should be discouraged."*
- Veterinarian 23: *"Appropriately used water sprayed not down the rima glottis, but on the aryepiglottic folds such that it stimulates a swallow reflex, expediates the process of obtaining 3 good swallows and improves animal welfare reducing the time of the exam.*"

- Veterinarian 13: "*Squirting water to stimulate swallowing is [a] different issue to hitting/ frightening horses. Only very small volumes used and no adverse effects experienced by horse when water used in responsible manner."*

- Veterinarian 40: "*I think it is essential that there is a period of unstimulated movements to enable the viewer to assess resting laryngeal movement, including in the period immediately post swallow. Too often we see 'waterboarding' where there is very frequent squirting with water*."

Participant responses related to the use of noise concluded that frightening the horse is unacceptable, but noise can be used as a distraction technique to improve safety and horse welfare during the endoscopy process:

- Veterinarian 40: *"Frightening or injuring the horse is not acceptable. Distracting the horse (playing animal noises, rustling wrappers, etc) as you would a child at the doctors, may be acceptable."*
- Veterinarian 23: "W*hen used appropriately acts as a distraction from the process rather than something to cause fear in the horse*."
- Veterinarian 32: "*I think the noise questions are limiting because sometimes talking to the yearlings and distracting them from the stress of the crush or crinkling a bandage in front*

of them similar to when a photo of a horse is taken at a show to attract attention is appropriate versus the horse jumping out the crush in fear."

Researcher response: The researchers acknowledge the question should have been divided into two, with one question asking about the use of noise and one asking about the use of water spray. We acknowledge that the question should have defined the type and endoscopist reasoning for use of noise, plus the amount/frequency of water usage when asking if this was not acceptable on animal welfare grounds.

At present, there are no available studies to determine if the frequency and amount of water sprayed can affect yearling laryngeal function grade, or compromise animal welfare, therefore no recommendations can be made at this time. However, until proven otherwise, it is reasonable to suggest that spraying frequent amounts of water through the rima glottidis and not allowing the horse to recover in between swallows, should be discouraged on animal welfare grounds.

○ Please click to continue to the next question.

Question 13: As per the guidelines, do you agree that a minimum of 3 distinct swallows should be recorded, which clearly show the degree of abduction of both arytenoid cartilages and allow evaluation of the whole larynx during swallowing?

Participant responses (n = 39):

- **Yes: 36 (92.3%)**
- **No: 3 (7.7%)**
- **Unsure: 0 (0%)**
- **Prefer not to say: 0 (0%)**

OUTCOME: Consensus achieved. A minimum of 3 distinct swallows should be recorded, which clearly show the degree of abduction of both arytenoid cartilages and allow evaluation of the whole larynx during swallowing.

○ Please click to continue to the next question.

Question 14: Which nostril/nasal passage do you use when performing pre-sales endoscopy?

Participant responses (n = 39):

- **Right: 14 (35.9%)**
- **Left: 20 (51.3%)**
- **Variable: 5 (12.8%)**
- **Prefer not to say: 0 (0%)**

OUTCOME: Consensus not achieved.

Researcher response: To our knowledge, there are no studies investigating the effect of nasal side on laryngeal function grade in yearlings, therefore we should refrain from making

recommendations at this time. Further research is warranted.

○ Please click to continue to the next question

Question 15: Do you use nasal occlusion when assessing laryngeal function during pre-sale endoscopy?

Participant responses (n = 39):

- **Yes: 5 (12.8%)**
- **No: 14 (35.9%)**
- **Variable: 20 (51.3%)**
- **Prefer not to say: 0 (0%)**

OUTCOME: Consensus not achieved.

Researcher response: To our knowledge, there are no studies investigating the use of nasal occlusion on laryngeal function grade in yearlings, therefore we should refrain from making recommendations at this time. Further research is warranted.

○ Please click to continue to the next question

Question 16: How should laryngeal function be graded if a horse shows variable degrees of laryngeal function during the same endoscopic examination?

Participant responses (n = 39):

- **Graded as the best laryngeal function grade observed: 10 (25.6%)**
- **Graded as the worst laryngeal function grade observed: 4 (10.3%)**
- **Graded as the most common (average) laryngeal function grade observed: (65.1%)**
- **Unsure: 0 (0%)**
- **Prefer not to say: 0 (0%)**

OUTCOME: Consensus not achieved.

Researcher response: All data presented on yearling endoscopy produced by our research team, was based on the average yearling laryngeal function grade seen on each endoscopic examination.

○ Please click to continue to the next question.

Question 17 (for vets working in Australia): Do you support a change in Australian pre-sales endoscopy protocol, to allow the examination to be performed prior to arrival at the sales complex, provided that it is still performed within 7 or fewer days before commencement of the sale?

Participant responses (n = 20), as vets working in NZ were excluded:

- Yes: 6 (30.0 %)
- No: 13 (65.0%)
- Unsure: 1 (5%)

OUTCOME: Consensus not achieved.

The majority of participants were supportive of keeping pre-sale scoping on complex in Australia, with reasoning including improved transparency, consistency in the endoscopic technique and essentially improved buyer confidence in the process:

- Veterinarian 23: "*Presale endoscopy is performed by vendors to improve buyer confidence. On complex exams provide more transparency to the process that has been perceived to be sub optimal*."
- Veterinarian 32: "*I think onsite is the best option- safe and reliable crush and techniques and quality tend to be more uniform."*

  Veterinarian 4: "*Probity is the issue*."
- Veterinarian 17: "*I believe it is in the vendor's/ purchaser's veterinarian's best interest to see a fully representative video as close to the sale date as possible on-site. This allows any pathology to be picked up pre-sale (post travel) reducing the incidence of surprise post-sale pathology and related issues."*

Reasonings for pre-sales examinations being performed on-farm included less stress on the yearlings, less time constraints and "*a safer environment for both horses and humans* (veterinarian 7)."

Participants working in New Zealand, also expressed support for a change in the New Zealand process back to on-complex scoping, to match the Australian process:

- Veterinarian 39: "*For the integrity of the Australasian marketplace we need uniformity, as it the same buying bench in both countries."*

○ Please click to continue

Thank you for participating in this delphi survey on yearling endoscopy. There are no further questions or survey rounds.

Once finalised, will email through a copy of the research paper to all participants.

If you have any further comments, please write them in the space below.

Powered by Qualtrics
