# Supplementary material for: Delphi consensus on Thoroughbred yearling sales endoscopy in Australasia
Source: Equine Vet J. 2026 Apr 14;58(5):1283–92. doi: 10.1002/evj.70164 (PMC13447881; doi:10.1002/evj.70164)
Supplement: Supplementary file 6 [file EVJ-58-1283-s006.pdf]

Survey S4

Delphi Round 3 - Results

Response rate: We continued to have excellent participant engagement in this survey round, with 36 out of 39 (92.3%) eligible participants completing the third Delphi survey.

Question 8: Considering the outcomes of this Delphi survey, do you support the use of a single risk table when evaluating yearling laryngeal function?

Round 3 question 8 responses (n = 36):

- Yes, I support the use of a single risk table: 34 (94.4%)
- No, I prefer the use of two separate risk tables: 0 (0%)
- Unsure: 1 (2.8%)
- Prefer not to answer: 1 (2.8%)

OUTCOME: Consensus achieved, as ≥ 75% of participants support the use of a single risk rating table.

Question 9: Do you agree with the risk table presented (table 4) for use when grading and communicating the risk of yearling laryngeal function?

Risk of developing laryngeal dysfunction that might impair race performance and/or require surgical intervention	Yearling laryngeal function grades[a]	Full arytenoid cartilage abduction is achieved at any time	Full arytenoid cartilage abduction is maintained on average for ≥ 0.2 secs[‡]	All arytenoid cartilage movements are symmetrical, or there is transient asynchrony/ flutter
Low	I, II.1	Yes	Yes	Yes
Low-moderate	II.2	Yes	Yes	No
Moderate	III.1	Yes	No	No
High	III.2, III.3, IV	No	No	No

Table 4: Agreed risk levels developed by expert consensus and descriptors of the three key yes/no decisions required to categorise yearling laryngeal function into the 4 risk levels.
[a]Havemeyer laryngeal function grading system (Dixon et al 2003).[2]
[‡]Definition of maintained abduction (Hardwick at al 2024).[1]

Round 3 question 9 responses (n = 36):

- Yes: 31 (86.1%)
- No: 1 (2.8%)
- Unsure: 2 (5.6%)
- Prefer not to answer: 2 (5.6%)

OUTCOME: Consensus achieved, as ≥ 75% of participants support the use of the risk table.

Qualitative analysis of participant feedback:

There was substantial participant support for the proposed single risk table:

- **Veterinarian 15**: "*Outstanding foresight in developing this as it addresses the divide between gradings that are poorly understood by clients and the common sale nomenclature of risk ratings. It also combines the 1 and 2.1 horses in the same group as they are essentially interchangeable both from a clinical endoscopic and performance/outcome standpoint.*"
- **Veterinarian 31**: "*Absolutely love this! I think this will make a huge positive difference.*"
- **Veterinarian 40**: "*I like that the questions are yes/no answers and are therefore very easy to come to a conclusion. It is objective and the table clearly provides a relative risk for each conclusion. Clients should be able to easily determine the risk they will be taking.*"

However, some participants felt that the proposed risk table could be confusing for clients and others were concerned that "*the risk rating should include reference to the signalment* (veterinarian 13)."

- **Veterinarian 3: "***I believe this is unduly complicated for a client to understand. It is of use to veterinarians to support their decisions in reaching a grading*."
- **Veterinarian 24**: "*My only concern is sex variation does not appear to be taken into account*."

There were mixed feelings about the layout of the table with it described as "*clear and concise* (veterinarian 17)", but also in need of condensing as "*there are too many words in first row of the table* (veterinarian 36)". Specific constructive feedback given to improve the table included:

- **Veterinarian 38: "***The last column is a little confusing; it would be easier for clients to understand if we described it as resting asymmetry present or not*."
- **Veterinarian 7**: "*Perhaps the wording could be "full abduction is achieved at some point during the examination" rather than any time?*"
- **Veterinarian 37**: "*While left side is 98% affected, should column 3 (from left) say "full abduction of both arytenoid cartilages" can be achieved. "At any time" seems unnecessary.*"

Researcher response:

Thank you for the feedback on the risk table.

To reduce the number of words in the first row, we have changed the title of column to **Risk†**, with further details provided in the table footnotes. We have also removed other superfluous words from the first row. Table 4 version 2 is below:

Risk†	Yearling laryngeal function grade‡	Full arytenoid cartilage abduction is achieved	Full arytenoid cartilage abduction is maintained on average for ≥ 0.2 secs§	Arytenoid cartilage movements are symmetrical, or there is transient asynchrony/flutter
Low	I, II.1	Yes	Yes	Yes
Low-moderate	II.2	Yes	Yes	No
Moderate	III.1	Yes	No	No
High	III.2, III.3, IV	No	No	No

Table 4: Agreed risk levels developed by expert consensus and descriptors of the three key yes/no decisions required to categorise yearling laryngeal function into the 4 risk levels.
†Risk of developing laryngeal dysfunction that might impair race performance and/or require surgical intervention.
‡Havemeyer laryngeal function grading system (Dixon et al 2003).[2]
§Definition of maintained abduction (Hardwick at al 2024).[1]

To address participant concerns about sex variation in recurrent laryngeal neuropathy, we would like to state the following:

The data utilised in our previous research, referenced within this Delphi study, were adjusted for sex, ensuring the results are interpretable for both males and females.[3,4] Recurrent laryngeal neuropathy has been characterized as a condition linked to extreme nerve length and is reported to affect males more frequently than females.[5,6] However, this disparity may stem from differences in body size—and consequently nerve length—between sexes, rather than the direct influence of sex itself.[7] Additionally, there are disparities in performance metrics between the sexes in normal race populations, as gelded males tend to have more race starts, greater career earnings, and extended career durations compared to females.[8,9] Moreover, a notably higher proportion of males were present in the prosthetic laryngoplasty group compared to the control group, which may reflect owner preferences to operate on gelded males and retire females to stud, rather than being solely attributable to a higher incidence of disease in males.[3] Ultimately, we believe it is up to the individual veterinarian to interpret the risk category for yearling laryngeal function, along with other risks of purchase (e.g. radiographic findings, phenotype), to determine the suitability for their client.

References

1. Hardwick JL, Ahern BJ and Franklin SH. A first step towards objective grading of equine laryngeal function. The Vet J, 2024; 308, 106259. https://doi.org/10.1016/j.tvjl.2024.106259.
2. Dixon, P., Robinson, E. and Wade, J.F. Consensus statements on equine recurrent laryngeal neuropathy. 2003. https://havemeyerfoundation.org/wp-content/uploads/2021/09/monograph11-Stratford-Monograph.pdf.
3. Hardwick JL, Ahern BJ, Crawford KL, Allen KJ and Franklin SH. Yearling laryngeal function in Thoroughbreds that underwent a laryngoplasty differs from controls. Equine Vet J. 2025; 57(2): 431–440. https://doi.org/10.1111/evj.14110
4. Hardwick JL, Ahern BJ, Crawford KL, Allen KJ, Anderson BH, Rose KJ and Franklin SH. Yearling laryngeal function grades II.2 and below are not associated with reduced performance. Equine Vet J. 2025. https://doi.org/10.1111/evj.14452
5. Goulden, B.E. and Anderson, L.J. (1981) Equine laryngeal hemiplegia part II: some clinical observations. *N Z Vet J* 29.
6. Draper, A.C.E. and Piercy, R.J. (2018) Pathological classification of equine recurrent laryngeal neuropathy. *J Vet Intern Med* 32, 1397–1409.
7. Boyko, A.R., Brooks, S.A., Behan-Braman, A., Castelhano, M., Corey, E., Oliveira, K.C., Swinburne, J.E., Todhunter, R.J., Zhang, Z., Ainsworth, D.M. and Robinson, N.E. (2014) Genomic analysis establishes correlation between growth and laryngeal neuropathy in Thoroughbreds. *BMC Genomics* 15.
8. Flash, M.L., Shrestha, K., Stevenson, M.A. and Gilkerson, J.R. (2023) National participation levels in the 2017–2018 Australian thoroughbred racing season. *Aust Vet J* 101.
9. Flash, M.L., Crabb, H.K., Hitchens, P.L., Firestone, S.M., Stevenson, M.A. and Gilkerson, J.R. (2022) Factors associated with racing performance and career duration for Victorian-born Thoroughbreds. *Aust Vet J* 100.
