# Supplementary material for: Delphi consensus on Thoroughbred yearling sales endoscopy in Australasia
Source: Equine Vet J. 2026 Apr 14;58(5):1283–92. doi: 10.1002/evj.70164 (PMC13447881; doi:10.1002/evj.70164)
Supplement: Supplementary file 7 [file EVJ-58-1283-s007.pdf]

Equine Veterinary Journal BEVA

Survey S1

Default Question Block

THE UNIVERSITY of ADELAIDE

PARTICIPANT INFORMATION SHEET

PROJECT TITLE: A delphi study to formulate expert consensus on the yearling sales endoscopy process in Australia and New Zealand

HUMAN RESEARCH ETHICS COMMITTEE APPROVAL NUMBER: H-2024-252

PRINCIPAL INVESTIGATOR: Professor Sam Franklin

STUDENT RESEARCHER: Dr Josie Hardwick

STUDENT'S DEGREE: PhD

Dear Participant,

You are invited to participate in the research project described below.

What is the project about?

This research project aims to generate a consensus opinion of a group of expert equine veterinarians on the Australian and New Zealand thoroughbred yearling endoscopy process and create professional guidelines. The study will involve 2-4 rounds of online surveys. After each round, the answers from all participants will be deidentified and used to determine the questions for the next round, until a consensus is reached. A consensus is defined as ≥ 75% of the participants agreeing on each statement.

Who is undertaking the project?

This project is being conducted by Josie Hardwick and will form part of a PhD at the University of Adelaide under the supervision of Professor Sam Franklin.

Why am I being invited to participate?

You are being invited as you are an equine veterinarian working in Australia and/or New Zealand. You have a minimum of 5 years' experience in endoscopic examinations of Thoroughbred yearlings, either on a thoroughbred stud farm, as a sales veterinarian performing pre- or post-sale examinations, or interpreting examinations for prospective yearling purchasers.

What am I being invited to do?

You are being invited to complete a series of online surveys on the yearling sales endoscopy process. The collective answers from all participants will be used to formulate the next round of questions. Your participation will be kept confidential, and all responses will be deidentified prior to data analysis. Survey

Are there any risks associated with participating in this project?

Whilst every effort will be taken to ensure your participation is kept confidential, there is a risk of your responses being identifiable to other participants.

What are the potential benefits of the research project?

The research may result in improved industry confidence in the Australian and New Zealand yearling sales endoscopy process, reduced variability between veterinarians when grading the same endoscopic examinations and improved understanding of the significance of differing degrees of yearling laryngeal function.

Can I withdraw from the project?

Participation in this project is completely voluntary. If you agree to participate, you can withdraw from the study at any time. However, due to the nature of the study, it will not be possible to withdraw any of your data that has already been deidentified and analysed.

What will happen to my information?

Confidentiality and privacy:

Your participation in this project and your responses will be kept confidential. All data submitted will be de-identified prior to analysis. While all efforts will be made to remove any information that might identify you, as the sample size is small, complete anonymity cannot be guaranteed. However, the utmost care will be taken to ensure that no personally identifying details are revealed.

Storage: All information and project records will be securely stored on an encrypted hard drive, accessible only to the research team and the data will be kept for a minimum of 5 years.

Publishing: Information collected will be analysed and results reported and publicised in veterinary journals, as part of Josie Hardwick's PhD thesis and presented at conferences. Only the summary data will be published, and participants will not be identified.

Sharing: A summary of the results will be made available to all participants. Deidentified data may be used in future research.

Your information will only be used as described in this participant information sheet and it will only be disclosed according to the consent provided, except as required by law.

Who do I contact if I have questions about the project?

Professor Sam Franklin Ph: 0883137931 Email: sam.franklin@adelaide.edu.au

Dr Josie Hardwick Ph: 0893607676 Email: josephine.hardwick@adelaide.edu.au

Has this study been approved by a Human Research Ethics Committee?

The study has been approved by the Human Research Ethics Committee at the University of Adelaide (approval number H-2024-252). This research project will be conducted according to the NHMRC National Statement on Ethical Conduct in Human Research 2023.

What if I have a complaint or any concerns?

Questions

If you have questions about the study, you can contact the research team:

Name: Dr Josie Hardwick

Phone: 0893607676

Email: josephine.hardwick@adelaide.edu.au

Insurance and Compensation

The study has been indemnified by The University of Adelaide which is enforceable by a participant to seek compensation in addition to your right to seek compensation under the common law through the legal system in legal proceedings.

Complaints

If you wish to speak with an independent person regarding concerns or a complaint about this study, the University's policy on research involving human participants, or your rights as a participant, please contact the Human Research Ethics Committee's Secretariat on:

Phone: +61 8 8313 6028

Email: hrec@adelaide.edu.au

Post: Research Services, Level 3, Rundle Mall Plaza, 50 Rundle Mall, ADELAIDE SA 5000

Any complaint or concern will be treated in confidence and fully investigated. You will be informed of the outcome.

If I want to participate, what do I do?

Select 'Click to continue to consent form'. Please read the consent form and click the 'I have read the above information and agree to participate in the study'.

Yours sincerely,

Dr Josie Hardwick, Professor Sam Franklin, Professor Ben Ahern and Dr Brian Anderson

○ Click to continue to the consent form.

THE UNIVERSITY of ADELAIDE

<u>Human Research Ethics Committee (HREC) – Consent Form</u>

1. I have read the Participant Information Sheet and agree to take part in the following research project:

Title: A delphi study to achieve expert consensus and develop professional guidelines on the yearling sales endoscopy process in Australia and New Zealand.

Ethics Approval Number: H-2024-252

2. I have had the project, so far as it affects me, and the potential risks and burdens fully explained to my satisfaction by the research worker. I have had the opportunity to ask any questions I may have about the project and my participation. My consent is given freely.

3. Although I understand the purpose of the research project, it has also been explained that my involvement may not be of any benefit to me.

4. I agree to participate in the activities outlined in the participant information sheet.

5. I understand my participation is voluntary and that I am free to withdraw at any time up until submission of each survey.

6. I have been informed that the information gained in the project may be published in a journal article/ PhD thesis/conference presentation.

7. I have been informed that in the published materials I will not be identified and any personal information that could identify me will not be divulged.

8. I consent for the use of my data by the same or other researchers in future research projects that are an extension of, or closely related to, the original project. I understand personal information that may identify me (e.g. name, email address) will be removed or changed before

it is shared with other researchers.

9. I understand my information will only be disclosed according to the consent provided, except where disclosure is required by law.

10. I am aware that I should keep a copy of this Consent Form and the Participation Information Sheet.

To give consent to participate in this study, please click the box below, then proceed to the survey.

○ I have read the above information and consent to participate in this study.

Question 1 relates to current concerns surrounding the yearling sales endoscopy process.

A copy of our recent study findings regarding yearling sales endoscopy are available for download here:

Hardwick et al 2024a stakeholder perceptions of thoroughbred yearling sales endoscopy in australia

Hardwick et al 2024b yearling laryngeal function grades ii.2 and below are not associated with reduced performance proof 1

Hardwick et al 2024c yearling laryngeal function in horses that underwent a prosthetic laryngoplasty

Hardwick et al 2024d a first step towards objective grading of equine laryngeal function

A yearling stakeholder engagement study (equine vets, breeders, purchasers of yearlings) identified three main concerns about the Australian sales endoscopy process:

- A lack of clarity surrounding the relationship between yearling laryngeal function and future performance, particularly surrounding those with grade 3 out of 5 (Lane 1993) laryngeal function.
- Variability between veterinarians (interobserver variability) when grading the same endoscopic examination.
- Variability in an individual horse's laryngeal function under different conditions (intrahorse variability). (Hardwick et al 2024a)

Question 1: What are <u>your</u> main concerns about the current yearling sales endoscopy process?

Please write response in space provided.

Question 2 relates to the laryngeal function grading system used at yearling sales.

The Proceedings of a Workshop on Equine Recurrent Laryngeal Neuropathy (Dixon et al 2004) containing the descriptions of the Havemeyer grading system (p96) and the Lane grading system (p24-25) are attached here: Havemeyer proceedings

The Havemeyer grading system and the Lane grading system (Lane 2003) are compared to each other in figure 1 below. Note that a grade 3 Lane is divided into two separate grades using the Havemeyer system (grades II.2 and III.1). The average proportion (%) of yearlings with each grade using both systems is also shown in red.

Lane grading system		Havemeyer grading system	
29.8%	1	I	29.8%
49%	2	II.1	49%
20.4%	3	II.2	16.5%
		III.1	3.9%
0.8%	4	III.2	0.8%
		III.3	0%
0%	5	IV	0%

Figure 1: Comparison between the Lane grading system used in the 2024 Conditions of Sale, compared to the Havemeyer grading system. The number in red represents the average proportion (%) of yearlings at sale with each grade (Hardwick et al 2024c).

Question 2: Considering recent findings (Hardwick et al 2024b, Hardwick et al 2024c), which grading system do you think should be used to evaluate yearling laryngeal function?

- ◯ Lane grading system (5-point scale)
- ◯ Havemeyer grading system (7-point scale)
- ◯ Neither

Question 3 relates to the pass/fail benchmark on a post-sale endoscopic examination at yearling sales.

In the 2024 Conditions of Sale for a post-sale endoscopic examination at Australian and New Zealand yearling sales, grades 4 and 5 laryngeal function (Lane 1993) 'fail' (do not meet the conditions of sale), whereas grades 1, 2 and 3 'pass' (meet the conditions of sale). Figure 2 highlights this pass/fail benchmark used on a post-sale endoscopic examination against the Havemeyer grading system.

Lane grading system	Havemeyer grading system
1	I
2	II.1
3	II.2
	III.1
4	III.2
	III.3
5	IV

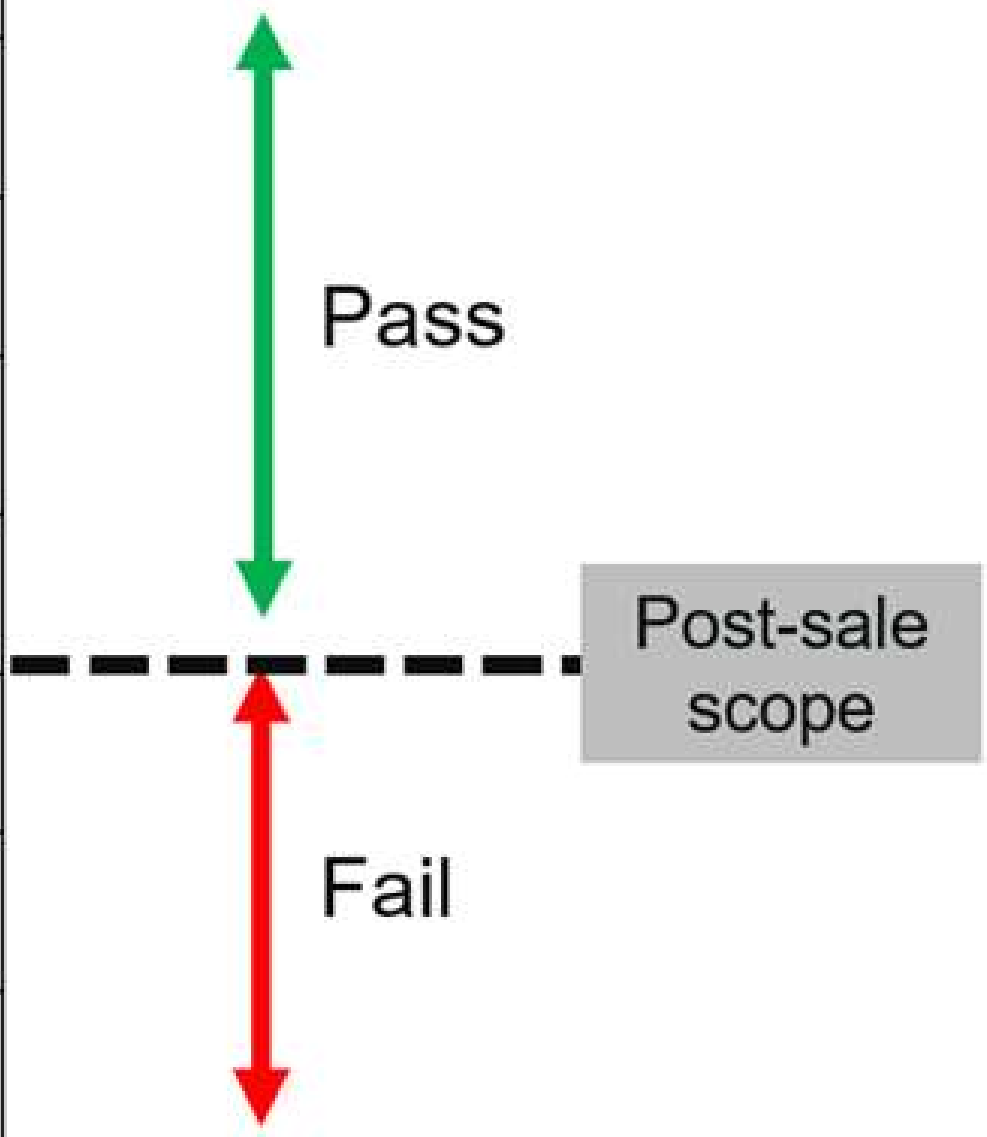

Figure 2: Comparison of the Lane grading system to the Havemeyer grading system. The hashed line represents the 2024 cut-off between laryngeal function grades that meets the conditions of sale ('pass', ≤ grade 3 Lane) and laryngeal function grades that do not meet the conditions of sale ('fail', ≥ grade 4 Lane).

Question 3: For post-sale endoscopy, do you agree that yearling laryngeal function ≤ grades III.1 should constitute a pass (meet the conditions of sale) and ≥ grades III.2 and above should constitute a fail (do not meet the conditions of sale)?

○ Yes, I agree that on a post-sale endoscopic exam, grades III.2 and III.3 should constitute a fail and ≤ grade III.1 should constitute a pass.

○ No, I believe the degree of laryngeal function that constitutes a fail on a post-sale examination should change.

○ Unsure.

Question 4 relates to the development of a risk rating categories for communicating the significance of each yearling laryngeal function grade around the time of sale.

A summary of two recent publications on yearling laryngeal function grade in terms of future race performance and the risk of prosthetic laryngoplasty surgery are listed below (Hardwick et al 2024b, Hardwick et al 2024c):

Grade I and II.1 yearling laryngeal function:

- No difference in race performance was identified between these grades
- There was no difference in risk of needing a prosthetic laryngoplasty between grades I and II.1.

Grade II.2 yearling laryngeal function:

- No difference in race performance was identified on average when compared to yearlings with grade I and II.1 laryngeal function.
- There was 3.3 times the risk of needing a prosthetic laryngoplasty, compared to yearlings with grade I laryngeal function.

Grade III.1 yearling laryngeal function:

- No difference in average total career earnings compared to yearlings with grade I/II.1 laryngeal function, however grade III.1's did earn significantly less ($35,076 less) than the average earnings for grade I/II.1's ($82,734) in their 4-year-old plus careers.
- There was 13.8 times the risk of needing a prosthetic laryngoplasty, compared to yearlings with grade I laryngeal function.

Grade III.2 yearling laryngeal function:

- Earned significantly less ($46,015 less) on average in their total career, compared to grade I/II.1 (average total career earnings of $110,923).
- Earned significantly less in their 4-year-old plus careers ($53,219 less) than yearlings with grade I/II.1 laryngeal function (average earnings of $82,734).
- There was 55.5 times the risk of needing a prosthetic laryngoplasty, compared to yearlings with grade I laryngeal function.

The above data was then used to model the mean probability of each yearling laryngeal function grade developing clinical recurrent laryngeal neuropathy (requiring a prosthetic laryngoplasty), based on a 10% overall prevalence of laryngeal dysfunction (presented by J. Hardwick at Bain Fallon 2024).

The mean probability of developing clinical recurrent laryngeal neuropathy needing a prosthetic laryngoplasty for each yearling laryngeal function grade (figure 3) was:

- Grade I/II.1: 6-7% probability (not statistically significant between grade I and II.1)
- Grade II.2: 14% probability
- Grade III.1: 32% probability
- Grade III.2: 42% probability*

(*Due to low numbers of yearlings with grade III.2 laryngeal function, statistical conclusions for this grade need to be made with caution).

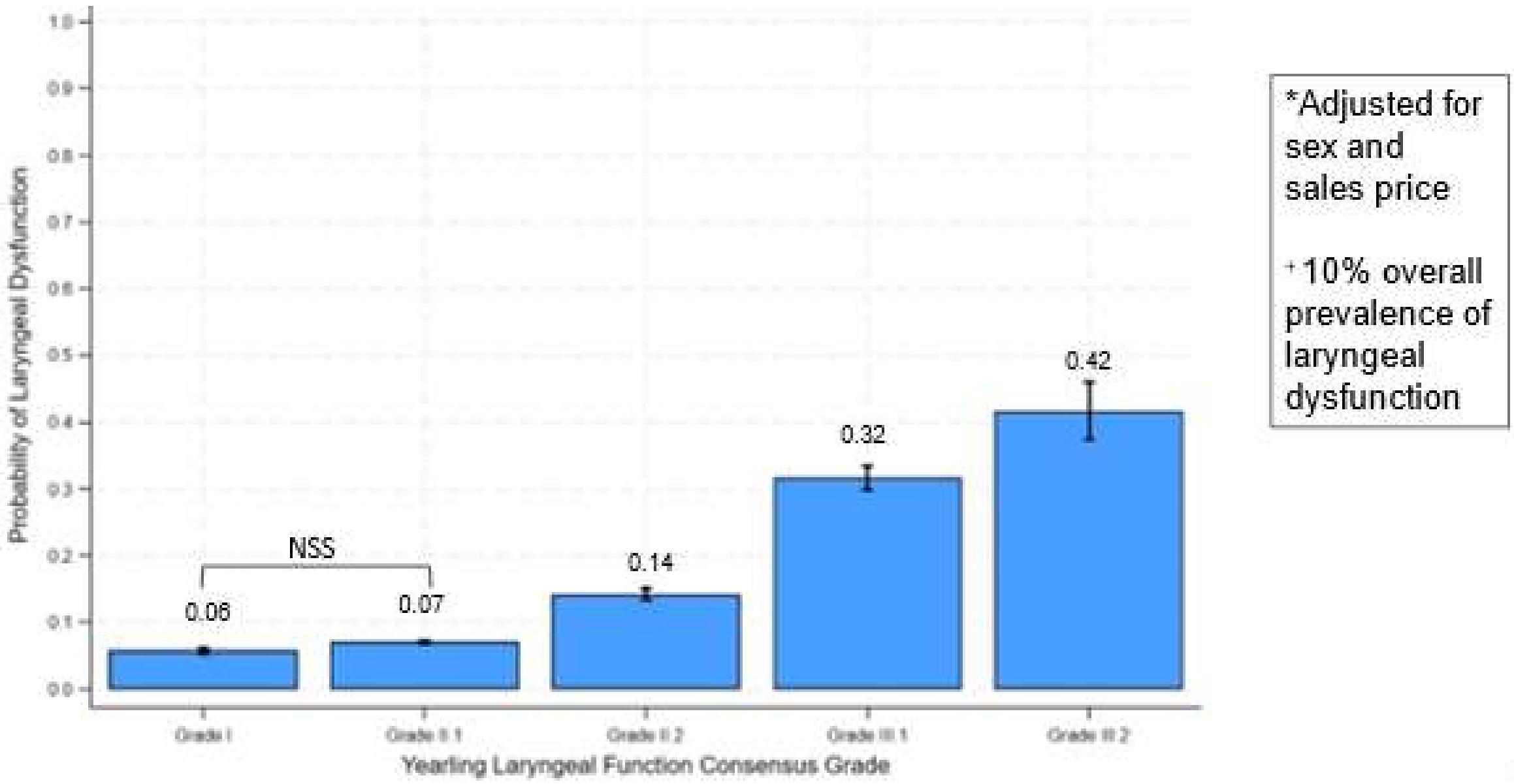

Figure 3: Modelled mean probability of each yearling laryngeal function grade needing a prosthetic laryngoplasty.

These results could be used to develop risk rating categories for each yearling laryngeal function grade, for equine veterinarians to use when communicating with vendors and purchasers of yearlings. This would be similar to the risk categories used when evaluating yearling radiographs (i.e. low-, moderate-, high-risk).

Question 4: Do you support the use of risk rating categories to evaluate yearling laryngeal function?

- ◯ Yes
- ◯ No
- ◯ Unsure

Question 5: Risk rating category: <u>Risk of reduced future performance</u>

For each yearling laryngeal function grade, please select your preferred risk rating category.

(The risk rating for developing clinical recurrent laryngeal neuropathy requiring a prosthetic laryngoplasty will be covered in question 7.)

	Low risk	Low-moderate risk	Moderate risk	Moderate-high risk	High risk
**Grade I**	○	○	○	○	○
**Grade II.1**	○	○	○	○	○
**Grade II.2**	○	○	○	○	○
**Grade III.1**	○	○	○	○	○
**Grade III.2**	○	○	○	○	○
**Grade III.3**	○	○	○	○	○
**Grade IV**	○	○	○	○	○

Question 6: Please explain your reasoning for the risk ratings given for each laryngeal function grade.

Write your comments in the space provided.

Question 7: Risk rating category: <u>Risk of developing clinical RLN and needing a prosthetic laryngoplasty</u>

For each yearling laryngeal function grade, please select your preferred risk rating category.

	Low risk	Low-moderate risk	Moderate risk	Moderate-high risk	High risk
**Grade I**	○	○	○	○	○
**Grade II.1**	○	○	○	○	○
**Grade II.2**	○	○	○	○	○
**Grade III.1**	○	○	○	○	○
**Grade III.2**	○	○	○	○	○
**Grade III.3**	○	○	○	○	○
**Grade IV**	○	○	○	○	○

Question 8: Please explain your reasoning for the risk ratings given for each laryngeal function grade.

Write your comments in the space provided.

Thank you for completing survey 1.

Please write any other comments and/or justifications for your answers in the space provided.

Powered by Qualtrics
